# AutA and AutR, Two Novel Global Transcriptional Regulators, Facilitate Avian Pathogenic *Escherichia coli* Infection

**DOI:** 10.1038/srep25085

**Published:** 2016-04-26

**Authors:** Xiangkai Zhuge, Fang Tang, Hongfei Zhu, Xiang Mao, Shaohui Wang, Zongfu Wu, Chengping Lu, Jianjun Dai, Hongjie Fan

**Affiliations:** 1 Key Lab of Animal Bacteriology, Ministry of Agriculture, Nanjing Agricultural University, Nanjing 210095, China; 2 Beijing Veterinary Research Institute, Chinese Academy of Agricultural Sciences, Beijing 100193, China; 3 Shanghai Veterinary Research Institute, Chinese Academy of Agricultural Sciences, Shanghai 200241, China

## Abstract

Bacteria can change its lifestyle during inhabiting in host niches where they survive and replicate by rapidly altering gene expression pattern to accommodate the new environment. In this study, two novel regulators in avian pathogenic *Escherichia coli* (APEC) were identified and designated as AutA and AutR. RT-PCR and β-galactosidase assay results showed that AutA and AutR co-regulated the expression of adhesin UpaB in APEC strain DE205B. Electrophoretic mobility shift assay showed that AutA and AutR could directly bind the *upaB* promoter DNA. *In vitro* transcription assay indicated that AutA could activate the *upaB* transcription, while AutR inhibited the *upaB* transcription due to directly suppressing the activating effect of AutA on UpaB expression. Transcriptome analysis showed that AutA and AutR coherently affected the expression of hundreds of genes. Our study confirmed that AutA and AutR co-regulated the expression of DE205B K1 capsule and acid resistance systems in *E. coli* acid fitness island (AFI). Moreover, phenotypic heterogeneity in expression of K1 capsule and acid resistance systems in AFI during host–pathogen interaction was associated with the regulation of AutA and AutR. Collectively speaking, our studies presented that AutA and AutR are involved in APEC adaptive lifestyle change to facilitate its infection.

The phenotypes of extraintestinal pathogenic *E. coli* (ExPEC) includes avian pathogenic *E. coli* (APEC), uropathogenic *E. coli* (UPEC), neonatal meningitis *E. coli* (NMEC), and septicemic *E. coli*^1^. The highly virulent APEC/ExPEC dominant O1:K1, O2:K1, and O18:K1 serotypes strains share significant genetic similarities and hold indistinguishable virulence features^2^,^3^,^4^. In recent years, due to significant mortality, drug resistance, economic losses, and zoonotic potential, the avian colibacillosis caused by APEC attracts more and more attention^4^,^5^. To infect the host extraintestinally, APEC requires adhesins to colonize the avian lungs. During adherence to lung tissues, host immune system can suppress and clear out APEC. Only highly virulent APEC can spread in bloodstream and cause fatal multisystemic infection^6^,^7^,^8^,^9^. APEC requires phagocytosis resistance or intracellular survival factors to evade and escape the lungs immune system^9^,^10^,^11^. It is well-known that the virulence factor K1 capsule facilitates APEC/ExPEC extraintestinal infection. K1 capsule of APEC/ExPEC is involved in survival and replication within non-phagocytosis cells, intracellular macrophage survival for disrupting host (phagocytosis) response modulation, and serum resistance to complement-mediated killing^12^,^13^,^14^,^15^. Interestingly, K1 capsule mutants of APEC/ExPEC appear to have higher adhesion rates during adherence on non-phagocytosis cells, but have lower intracellular survival within host cells, implying that presence of polysaccharide capsule might shield the virulence roles of short bacterial adhesins^15^,^16^,^17^.

Bacteria can switch lifestyle to inhabit in different host niches by rapidly altering its gene expression levels to accommodate the new environment^18^. Adaptive lifestyle switch relies on transcriptional regulators to cooperatively alter gene transcription. They can directly activate or repress the genes expression by binding to promoter region of targeted genes^19^,^20^,^21^. Transcriptional regulators contain typical LuxR/GerE family regulators in a variety of organisms, which harbor the DNA-binding helix-turn-helix (HTH) motif to conduct the binding to target promoter sequence^19^,^20^,^22^. The DNA-binding actions of regulators are controlled and modified by its N-terminal kinase phosphorylation or autoinducer-binding domains^19^,^20^,^23^,^24^.

In this study, the novel APEC virulence regulatory networks were presented that AutA and AutR played roles in APEC adaptive lifestyle switch by coordinately regulating the expression of AT adhesins, K1 capsule, and acid resistance systems to facilitate APEC infection.

## Results

### Identification of two putative transcriptional regulators in APEC UpaB cluster

**Figure 1:**
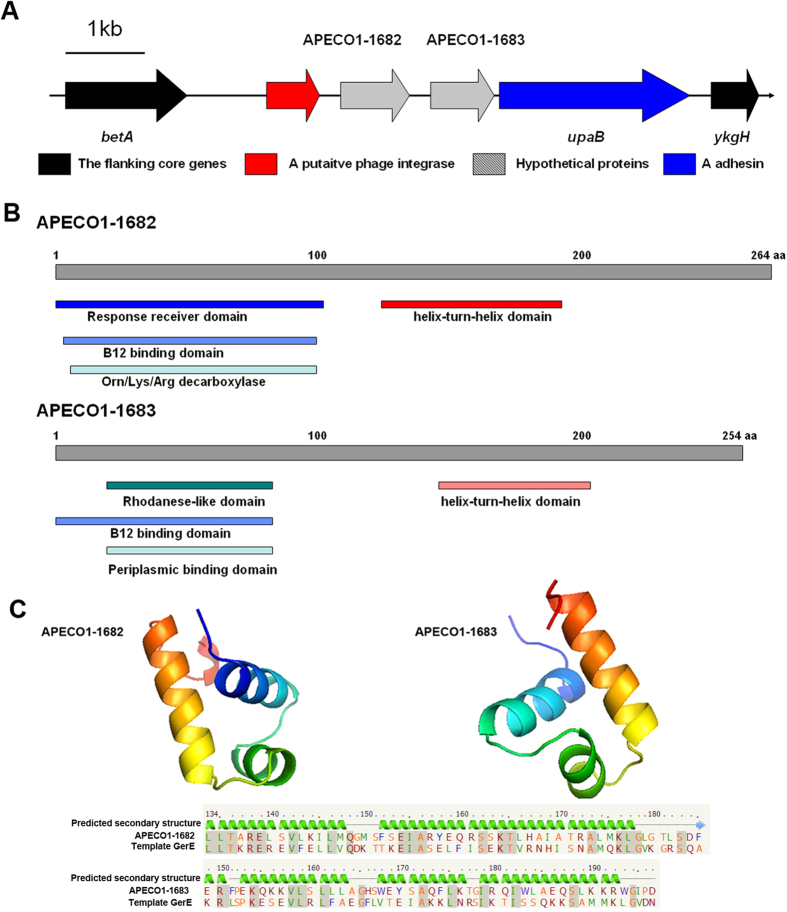
Identification of two putative transcriptional regulators in UpaB cluster. (**A**) Chimeric feature and genetic context of UpaB cluster. The UpaB cluster was inserted in the *E. coli* core genes *betA* and *ykgH* in DE205B and contained four ORFs. The arrows and its direction represented the direction of transcription for proteins encoded by the four genes. (**B**) Schematic illustration of the conserved domains for two transcriptional regulators encoded by *APECO1-1682/1683*. Indicated are the predicted domains of APECO1-1682 and -1683 harbored the typical HTH motif and several putative enzyme domains for response signal receiver. (**C**) The sequence alignment and secondary structure prediction of the HTH motifs of APECO1-1682 and -1683 compared to LuxR family regulator GerE. The structure of HTH motifs of APECO1-1682 and -1683 were predicted as three-helical bundle structure using Phyre^2^ aginst the crystal structure of regulator GerE, respectively.

Recent studies proved that the autotransporter adhesin UpaB, associated with APEC and UPEC pathogenicity^25^,^26^, is located in GI-4 of APEC O1. It is also called UpaB cluster in APEC DE205B. UpaB cluster was inserted between *betA* and *ykgH* of *E. coli* core genes in DE205B (Fig. 1A). The UpaB cluster included additional three genes, which were a putative phage integrase gene *APECO1-1681* and two hypothetical ORFs *APECO1-1682* and *APECO1-1683*. The G+C content of this cluster is 43.08%, which was significantly lower than the average percentage (~50%) of *E. coli* genomes (Fig. 1A). Since a phage integrase in this cluster and its lower G+C content, horizontal gene transfer might be involved in the presence of UpaB cluster in *E. coli* genome. Bioinformatic analysis showed that two genes *APECO1-1682/1683* might encode two transcriptional regulators. The protein motif search showed that the APECO1-1682 and APECO1-1683 might harbor the typical HTH motif and several putative enzyme domains (Fig. 1B). The sequence alignment showed that the HTH motifs of APECO1-1682 and -1683 shared 37% and 24% identity with LuxR family regulator GerE, respectively (Fig. 1C). As shown in Fig. 1C, the structure of HTH motifs of APECO1-1682 and -1683 were predicted as three-helical bundle structures using Phyre^2^ against the crystal structure of regulator GerE, respectively. *APECO1-1682*/*1683* did not show high homology to any identified regulator genes and were not variants of response regulator genes, suggesting that APECO1-1682/1683 are novel LuxR/GerE family regulators.

### The UpaB cluster was high present among APEC ECOR B2 and D strains

The presence of UpaB cluster among 347 APEC isolates in China was determined via multiplex PCR and showed isolates (36.0%) containing this cluster. The ECOR analysis of APEC isolates harboring UpaB cluster were determined: 15.6% (23/147) of group A, 23.2% (19/82) of group B1, 74.4% (32/43) of group B2, and 68.0% (51/75) of group D. This cluster was present in the all highly virulent APEC O1:K1 and O2:K1 strains (total 16 strains in B2 isolates)^4^.

### The novel regulators AutA and AutR regulated expression of adhesin UpaB in APEC DE205B *in vitro*

The UpaB expression is highly induced during APEC infection, suggesting that the putative regulators might affect the UpaB expression. RNA level of four ORFs in UpaB cluster were similar to that of housekeeping gene *dnaE* (Figure S1). Furthermore, the co-transcription test for intergenic regions was performed to assess whether the four ORFs of UpaB cluster was located one operon. Our data showed that transcription among three intergenic regions wasn’t detected (Figure S1). Thus, the four ORFs of UpaB cluster acted as single transcriptional units.

**Figure 2:**
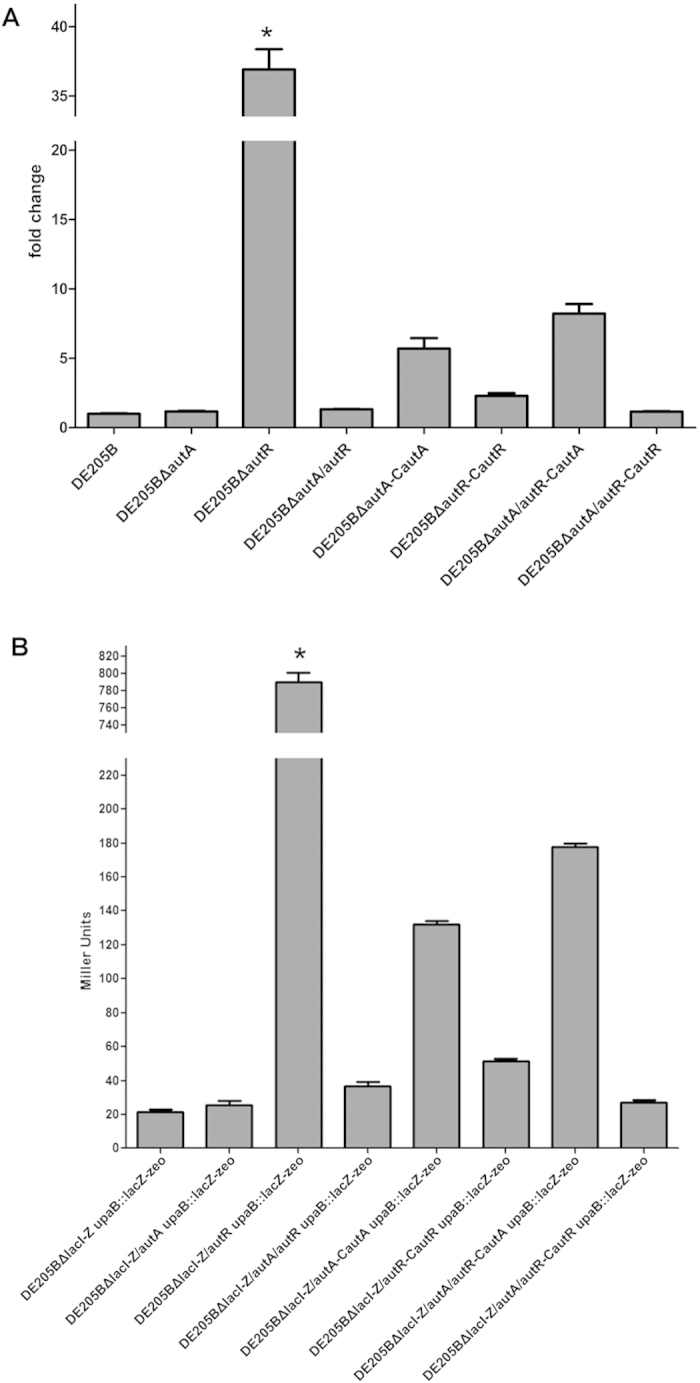
The AutA and AutR regulated the expression of UpaB in DE205B *in vitro*. (**A**) Quantification of the UpaB expression among DE205B mutants and complemented strains. The transcription of *upaB* gene among DE205B variants was determined by qRT-PCR. Three replicates were normalized to the transcription of housekeeping gene *dnaE*, and the fold change of *upaB* transcription among DE205B variants relative to the DE205B was calculated with the ΔΔ*C*_*T*_ method. (**B**) The β-galactosidase activity of each *upaB*::*lacZ* transcriptional fusion among DE205B variants was assayed by the Miller assay. The *upaB* transcriptional change was indirectly evaluated by the determination of β-galactosidase activity (in Miller units) among DE205B variants. Statistical significance analysis was performed using two-way ANOVA (*P < 0.05).

Single deletion of *APECO1-1683* led to a substantial increase (about 36.9-fold) in *upaB* transcription *in vitro* (*P* < 0.01), and the single deletion of *APECO1-1682* had no obvious effect on *upaB* transcription (Fig. 2A). However, compared with individual knockout DE205BΔ*1683*, dual deletion of *APECO1-1682/1683* genes led to a decreased *upaB* transcription, which was close to DE205B (Fig. 2A). These results showed that APECO1-1682 and APECO1-1683 might act as transcriptional regulators and co-regulate UpaB expression of DE205B. Here, we designated these two novel regulators as AutA (APEC UpaB Transcriptional Activator; APECO1-1682) and AutR (APEC UpaB Transcriptional Repressor; APECO1-1683). Moreover, overexpression of AutA or AutR partially complemented the effect of *autA* and *autR* deletion on *upaB* transcription (Fig. 2A). Overexpression of AutA in complemented DE205BΔ*autA-*C*autA* and DE205BΔ*autA/autR*-C*autA* exhibited 5.7–fold and 8.2–fold increase in *upaB* transcription compared with DE205B, respectively (*P* < 0.01) (Fig. 2A). Overexpression of AutR in DE205BΔ*autR-*C*autR* showed that the UpaB expression was restored to the similar level of DE205B. Overexpression of AutR in DE205BΔ*autA/autR*-C*autR* did not reduce UpaB expression, suggesting that AutR might directly suppress the activating effect of AutA on UpaB expression.

Chromosomal *upaB::lacZ-zeo* transcriptional reporter fusion was constructed in the mutant DE205BΔ*lacI-Z* (Figure S2). β-galactosidase activities of these mutants and complemented strains were performed. As expected, *lacZ* expression was activated in the presence of AutA. The AutR was identified as a repressor of P_*upaB*_ transcription due to suppression role in AutA activity. In summary, our data suggested that AutA acted as transcriptional activator, while AutR was a transcriptional repressor to co-regulate P_*upaB*_ transcription of *upaB* and *upaB::lacZ-zeo* genes in DE205B variants.

### MBP::AutA and MBP::AutR directly bound the P_*upaB*_ promoter region DNA and altered the *upaB* transcription *in vitro*

**Figure 3:**
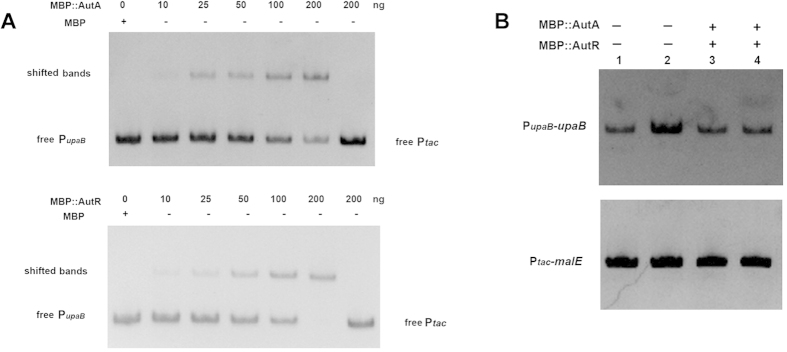
MBP::AutA and MBP::AutR directly binding the P_*upaB*_ promoter region and altering the *upaB* transcription *in vitro*. (**A**) Non-radioactive EMSA detecting the band shift of P_*upaB*_ promoter region. P_*upaB*_ DNA fragment (200 bp) and the negative control DNA fragment (200bp for *upaB* coding region) were amplified by PCR and used as EMSA probes. EMSAs were conducted by adding increasing amounts of MBP::AutA (up panel) and MBP::AutA (down panel) fusion proteins in each reaction mixture. The purified His-MBP fusion protein acted as the negative control for EMSA tests. The gels were stained with 1× SYBR Gold nucleic acid solution. (**B**) *In vitro* transcription assays to determine the effect of AutA and AutR on *upaB* transcription. The amount of RT-PCR products for both P_*upaB*_-*upaB* (upper panel) and P_*tac*_-*malE* (lower panel) (control) with or without adding MBP::AutA and MBP::AutA fusion proteins in each reaction mixture. The RT-PCR product directly corresponds to the transcription level of the corresponding RNA synthesized *in vitro*.

MBP::AutA and MBP::AutA fusion proteins were successfully expressed and purified in *E. coli* (Figure S3). The electrophoretic mobility shift assay (EMSA) on the *upaB* promoter DNA P_*upaB*_ was performed. As shown in Fig. 3A, both the purified proteins MBP::AutA and MBP::AutR were able to shift the P_*upaB*_ promoter DNA, but not the control fragment. The results demonstrate that regulators AutA and AutR could directly bind to the P_*upaB*_ promoter.

To determine how AutA and AutR activate or suppress *upaB* transcription, the *in vitro* transcription assays were performed. The transcription level of P_*upaB*_-*upaB* and P_*tac*_-*malE* was determined by RT-PCR. As shown in Fig. 3B, the transcription level of P_*upaB*_-*upaB* was increased by 9.2-fold in the presence of AutA (*P* < 0.01). Although MBP::AutR directly bound the P_*upaB*_ promoter region DNA, the presence of AutR had no effect on the transcription of P_*upaB*_-*upaB*. However, transcription level of P_*upaB*_-*upaB* in the presence of both MBP::AutA and MBP::AutR was lower than the transcription level in the presence of only MBP::AutA and close to the reaction without adding MBP::AutA and MBP::AutR (Fig. 3B). Our results concluded that AutA could activate the transcription P_*upaB*_-*upaB in vitro*. AutR could directly suppress the activating effect of AutA on UpaB expression and indirectly inhibited the P_*upaB*_-*upaB* transcription under the presence of AutR.

### Transcriptome analysis

**Figure 4:**
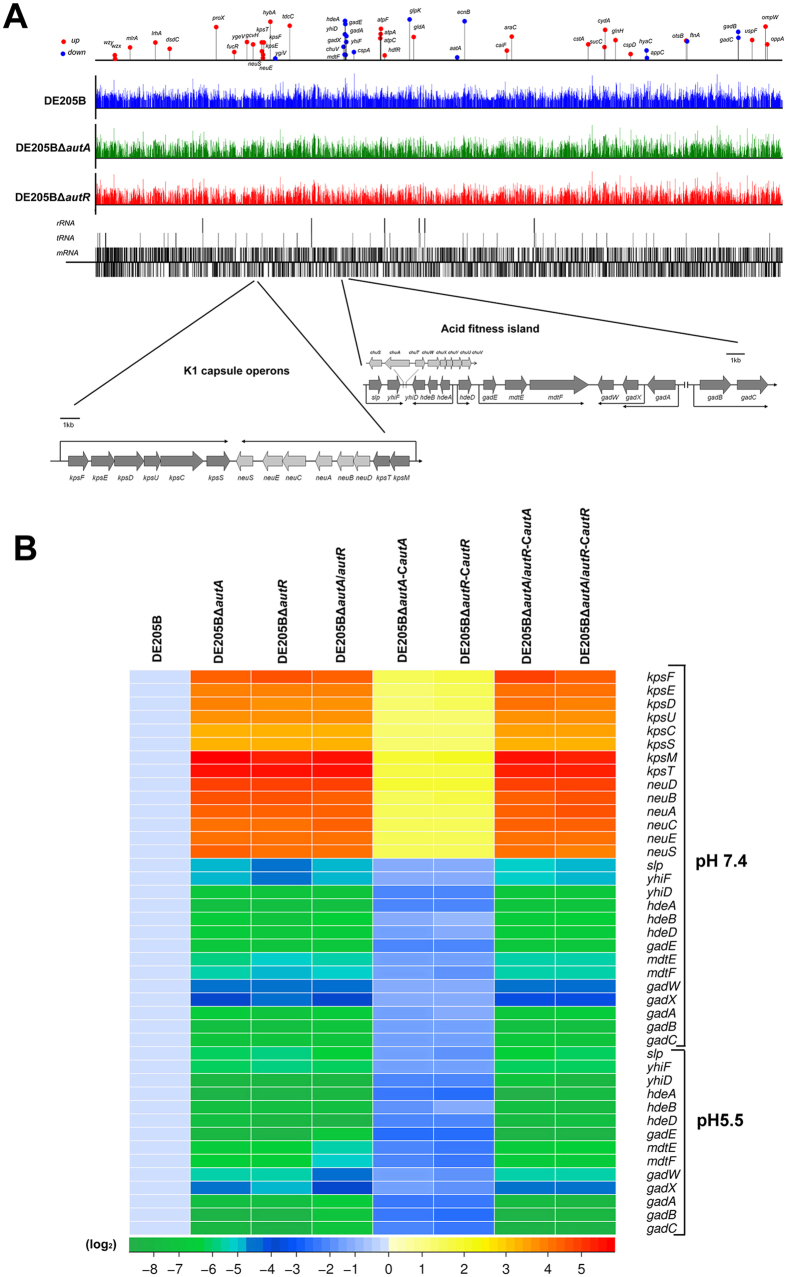
The genes transcription regulated by AutA and AutR. (**A**) An overview of global gene expression profiles of DE205B, DE205BΔ*autA*, and DE205BΔ*autR* at mid-logarithmic phase. The cDNA reads of DE205B and mutants for genome-wide landscape were adjusted to same scale, mapped to and plotted as log_10_^FPKM^ values over the IMT5155 genome. Vertical bars (grey) indicate gene clusters for mRNA, tRNA, and rRNA, and (up) and (down) indicate genes for forward and reverse strands of IMT5155 genome. The highlighted 50 genes for obviously differential peaks were plotted in the global expression map. The genetic context of K1 capsule determinant for two operons (*kpsFEDUC* and *kpsMTDBACES*)^28^ and the acid fitness island (AFI) for five operons, including *slp-yhiF, hdeAB-yhiD, gadE-mtdEF, gadXW* and *gadAX*^27^,^49^, were represented by arrows in forward and reverse direction for genome position. (**B**) The heat map summarized the transcription changes of acid resistance systems and K1 capsule determinant among mutants and complemented strains compared to that of DE205B cultured in LB (pH 7.4). Furthermore, the transcriptional level of Gad system for AFI among mutant/complemented strains and DE205B was determined in the pH 5.5 cultured conditions. RT-PCR data represent mean relative expression of three biological replicates.

The cDNA reads of DE205B and mutants for genome-wide landscape were adjusted to same scale, mapped to and plotted as log_10_^FPKM+1^ values over the IMT5155 genome (Fig. 4A). The RNA-seq analysis showed that mutants DE205BΔ*autA* and DE205BΔ*autR* shared similar genomic expression profiles except for UpaB expression (Fig. 4A). Transcription levels of 223 genes in DE205BΔ*autA* and DE205BΔ*autR* exhibited changes (>2-fold, *p* value <0.05) compared with DE205B. Except for the *gadBC* operon, the most significantly down-regulated (near 2^7^-fold) genes focused on the acid fitness island (AFI) in *E. coli*^27^ (*P* < 0.01) (Table S1; Fig. 4A). Moreover, the two operons of K1 capsule determinant encoding synthesis and transport proteins of *E. coli* K1 polysialic acid capsule were obviously up-regulated in *autA* and *autR* deletion mutants^28^ (Table S1; Fig. 4A).

RT-PCR of 50 genes among mutants and DE205B was conducted to validate the RNA-seq analysis. The quantitative PCR result (Table S2) showed the similar tendency to RNA-seq analysis. For detailed descriptions on transcriptome analysis, see Text S1 in the supplemental information.

### AutA and AutR co-regulated the expression of acid resistance systems in AFI and K1 capsule *in vitro*

For further identify the function of AutA and AutR, RT-PCR was performed on the genes encoding K1 capsule and acid resistance systems was conducted among mutant/complemented strains and DE205B cultured in LB (pH 7.4). Heat maps indicated the transcription changes of acid resistance systems and K1 capsule determinant among mutants compared to that of DE205B (Fig. 4B). Moreover, the transcriptional level of acid resistance systems and K1 capsule determinant in complemented DE205BΔ*autA-*C*autA* and DE205BΔ*autR-*C*autR* was restored to the similar level of DE205B. The transcription changes of acid resistance systems and K1 capsule determinant in DE205BΔ*autA/autR* was close to the level of these genes in DE205BΔ*autA* and DE205BΔ*autR*. However, transcriptional level of these genes in single complemented DE205BΔ*autA/autR*-C*autA* and DE205BΔ*autA/autR*-C*autR* were still maintained at the original level of DE205BΔ*autA/autR*. Furthermore, the transcriptional level of acid resistance systems among mutant/complemented strains and DE205B was determined in the pH 5.5. The transcription changes of acid resistance systems and K1 capsule determinant among mutant/complemented strains (cultured in pH 5.5) was similar to that in pH 7.4 (Fig. 4B). These results suggested that AutA and AutR coherently activated the transcription of Gad acid resistance system and repressed the transcription of K1 capsule determinant, and any deletion could cause the loss of the co-control and regulatory pathways.

**Figure 5:**
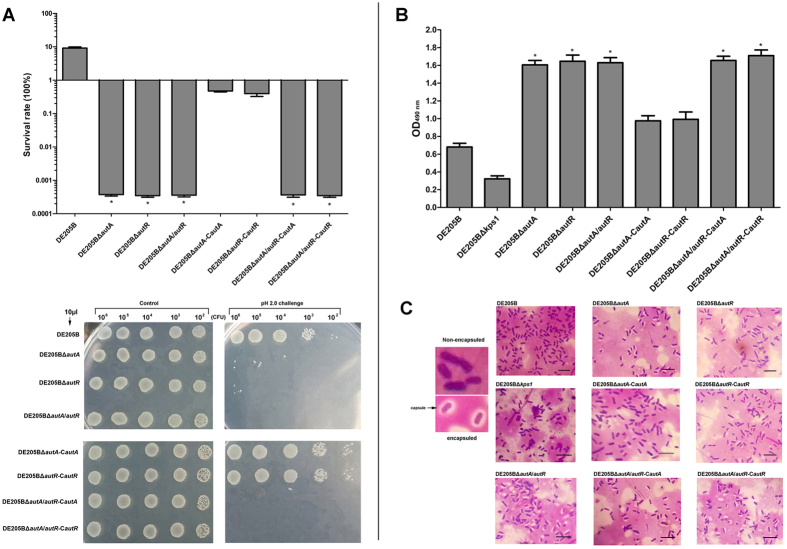
The effects of AutA and AutR on acid resistance and K1 capsule antigen of DE205B phenotype. (**A**) Acid resistance assays were performed to determine the survival rates among mutants and complemented strains compared to that of DE205B. Logarithmic growing *E. coli* cells were exposed to pH 2.0 for 2 h. Percent survival for bacterial acid resistance was measured by plate counting with three biological replicates. In parallel, tenfold serial dilutions of bacterial cells exposed to acid challenge were spotted onto LB agar plates. The pH 7.4 treated cells were used as a control. Statistical significance analysis was performed using one-way ANOVA (*P < 0.01). (**B**) Detection of K1 capsule expression of DE205B and several variants. The K1 capsule-specific antiserum was used to conduct the ELISA to detect the capsule antigen. The ELISA result of DE205BΔ*kps1* mutant strain acted as the negative control. Statistical significance analysis was performed by the Student *t* test (*P < 0.01). (**C**) To support the accuracy of the ELISA results, the microscopic observation to detect the K1 capsule among DE205B and variants. After stained, the K1 capsule of bacteria was colorless, and the encapsulated or nonencapsulated *E. coli* was indicated. DE205BΔ*kps1* mutant strain acted as the negative control.

Acid resistance assays were performed to determine the effects of AutA and AutR on APEC phenotype. Survival rates of DE205BΔ*autA*, DE205BΔ*autR*, and DE205BΔ*autA/autR* were significantly lower than that of positive control DE205B and exhibited a decrease by four orders of magnitude (*P* < 0.01) (Fig. 5A). Meanwhile, the complemented DE205BΔ*autA-*C*autA* and DE205BΔ*autR-*C*autR* increased the survival rates and reached to the level of DE205B. However, the survival rates of single complemented strains were still low (Fig. 5A). The K1 capsule antigen among mutant/complemented strains was tested by ELISA, and K1 capsule production among three mutants and two single complemented strains reflected a markedly increased expression than DE205B and DE205BΔ*kps1* mutant (deletion of *kpsFEDUC*) for negative control (Fig. 5B). The ELISA assay on K1 capsule antigen of complemented strains (DE205BΔ*autA-*C*autA* and DE205BΔ*autR-*C*autR*) showed similar optical density (OD_490_) to DE205B (Fig. 5B). Furthermore, the K1 capsule among these strains was stained and examined under the microscopy. Compared with the DE205B and the control DE205BΔ*kps1*, the colorless K1 capsule among *autA* and *autR* mutants were detected, and the K1 capsule examination among these strains using microscopy was consistent with treads for the ELISA result detecting K1 capsule antigen (Fig. 5C). The acid resistance test and the detection of K1 capsule further supported that the regulation roles of AutA and AutR were associated with acid tolerance and K1 capsule biosynthesis in *E. coli*.

### The phenotypic heterogeneity in expression of K1 capsule, acid resistance systems, and regulators AutA and AutR during DE205B infection

**Figure 6:**
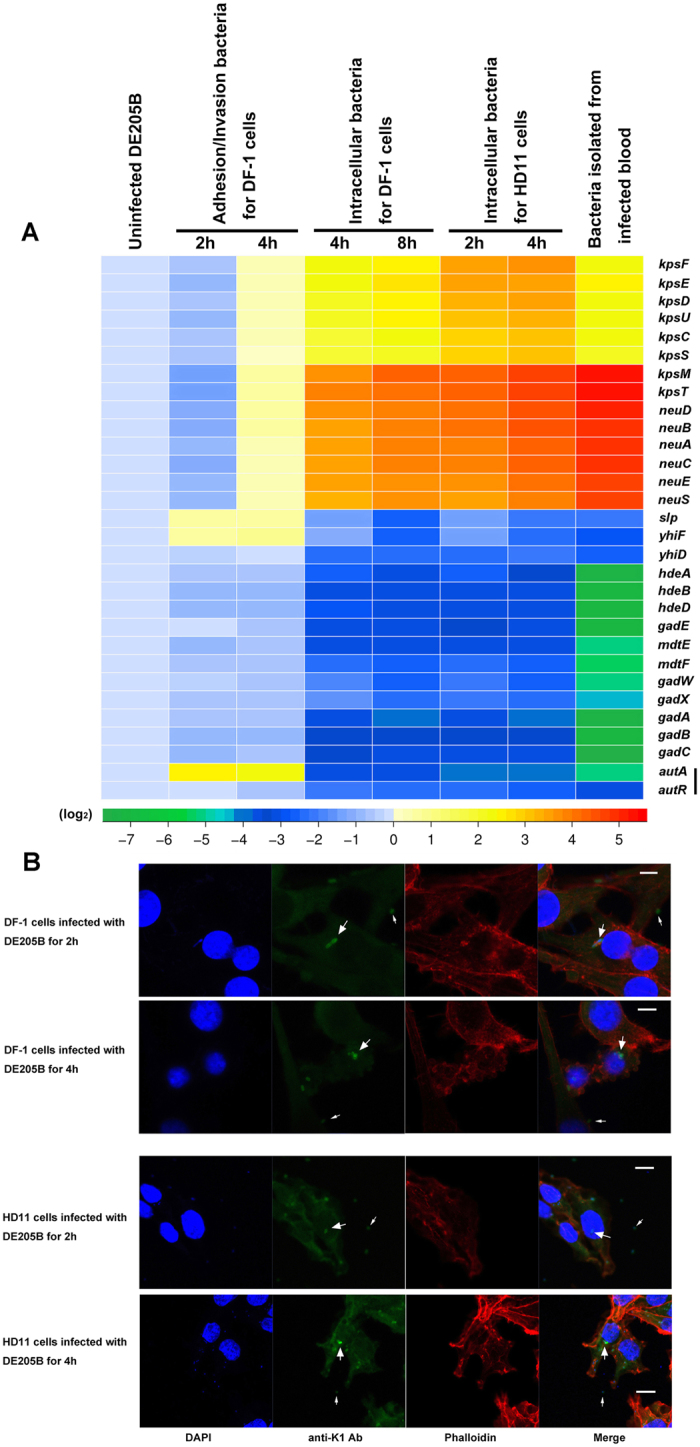
The phenotypic heterogeneity in expression of K1 capsule, acid resistance systems, and regulators AutA and AutR during DE205B infection. (**A**) The heat map summarized the transcription changes of acid resistance systems, K1 capsule determinant, and regulators (AutA and AutR) during DE205B infection by RT-PCR. The total RNA of DE205B were isolated from infected DF-1 cells, infected HD11, and blood for infected ducks at specific time points. RT-PCR data for heat map represent mean relative expression of three biological replicates. (**B**) Detection of K1 capsule expression during DE205B infection by immunofluorescence assay to support the accuracy of the above RT-PCR results. After the binding of K1 capsule-specific antibody, K1 capsule were visualized by FITC-labeled goat anti-rabbit secondary IgG, and observed using the confocal laser scanning microscope. The infected DF-1 and HD11 cells were incubated with DAPI and Phalloidin (actin stain; TRITC conjugated) to distinguish cell boundaries. Scale bar = 10 μm. Thick and thin arrows highlighted intracellular and extracellualr (adhesive) bacteria, respectively. The experiment was conducted three times, and the representative fields of observation are shown for each condition.

RT-PCR was used to determine the genes transcriptional level of K1 determinant, acid resistance systems in AFI, and *autA*/*autR* (relative to *dnaE*) during APEC infection *in vitro*. For DE205B adherence to and invasion of DF-1 cells, the total RNA (including all the bacteria interaction with cells) was acquired at two infection time points (2h and 4h). RT-PCR result showed that transcription level of each gene in K1 operons did not differ from the routinely cultured DE205B (negative control) (*P* ≥ 0.05) (Fig. 6A).

Since K1 capsule facilitates *E. coli* intracellular survival and replication, we speculated that the transcriptional level of K1 capsule determinant in intracellular DE205B might be higher than that of extracellular bacteria. Therefore, we performed the RT-PCR analysis on intracellular bacteria after gentamicin incubation to kill extracellular bacteria. The result showed the transcriptional level of K1 capsule genes in intracellular DE205B after infection for 4h and 8h were significantly increased than that of the control (*P* < 0.01)(Fig. 6A). Furthermore, immunofluorescence assay was conducted to examine the heterogeneous expression of surface K1 capsule. The infected DE205B (adherence to and invasion of DF-1 cells) were stained with anti-K1 Ab, and then DF-1 cells were fixed and incubated with DAPI and Phalloidin to differentiate between intracellular and extracellular bacteria. At time points (2 h and 4 h), the immunofluorescence signal of intracellular DE205B in DF-1 cells was higher than that of extracellular bacteria (Fig. 6B).

For entry, intracellular survival, and replication of DE205B in HD11 cells, cells were infected with the bacteria for 1h and 3h, followed gentamicin treatment for 1h, respectively. The RT-PCR result showed that transcriptional level of K1 genes in intracellular bacteria after interaction with HD11 cells for 2 h and 4 h were increased significantly than that of the control, respectively (*P* < 0.01)(Fig. 6A). Immunofluorescence assay showed that the signal of intracellular DE205B in HD11 cells was higher than that of extracellular bacteria at 2h and 4h, respectively (Fig. 6B). It is known that K1 capsule is involved in APEC serum resistance; we next tested the expression of K1 capsule in DE205B infected blood under duck sepsis. The transcriptional level of the genes in K1 operons was also increasingly expressed compared to the control (*P* < 0.01) (Fig. 6A).

Meanwhile, the transcriptional level of acid resistance systems in AFI, *autA*, and *autR* was measured. Our data showed that the transcriptional levels of these genes were downregulated for DE205B within DF-1/HD11 cells and isolated from duck blood *in vivo* (*P* < 0.01), accompanied with increasing expression of K1 capsule-related genes (Fig. 6A). The *autA* and *autR* genes in DE205B appeared a differential expression under different infection conditions. For DE205B adherence to and invasion of DF-1 cells for 2 h and 4 h, transcription level of *autA* was upregulated for 4.8–fold and 4.3–fold compared with the control, respectively (*P* < 0.01) (Fig. 6A). The transcription level of *autR* had no change significantly compared with the control (*P* ≥ 0.05). Moreover, the transcription level of *autA* and *autR* for intracellular DE205B in DF-1/HD11 cells and isolated from infected duck blood was all decreasing expressed compared with the control (*P* < 0.01) (Fig. 6A). Based on AutA and AutR co-regulating (activating or repressing) genes encoding K1 capsule and Gad acid resistance system *in vitro*, our results suggested that a heterogeneous expression pattern of K1 capsule and acid resistance systems during DE205B infection *in vivo* might be regulated by AutA and AutR for reciprocal phenotype regulation under host-induced stimuli.

### AutA and AutR contributed to DE205B phenotype fitness

**Figure 7:**
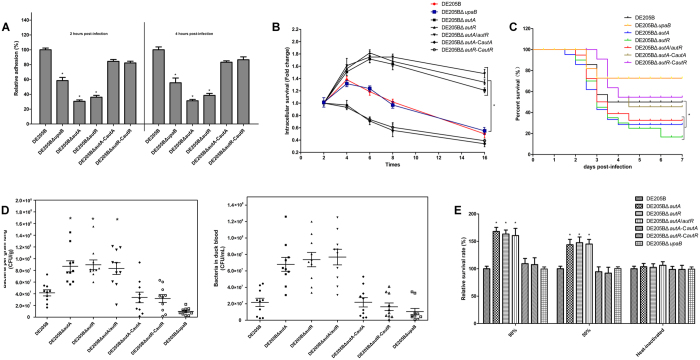
AutA and AutR contributed to DE205B phenotype fitness for intracellular survival, serum resistance, colonization in duck lungs, and sepsis *in vivo*. (**A**) DE205BΔ*autA* and DE205BΔ*autR* decreased its adherence on DF-1 cells for 2 h and 4 h post-infection compared with the control strains DE205B and DE205BΔ*upaB*. Statistical significance analysis was performed using one-way ANOVA (*P < 0.01). (**B**) The *autA* and *autR* mutants enhanced its intracellular survival within HD11 cells compared with the control strains DE205B and DE205BΔ*upaB in vitro*, as fold change in bacterial number at time points (4 h, 6 h, 8 h, and 16 h) relative to initial number of intracellular bacteria for 2 h. The two-way ANOVA was performed for survival assays (*P < 0.05). (**C**) To determine the effect of *autA* and *autR* loss on APEC virulence for duck model. The *autA* and *autR* mutants enhanced the survival/mortality rates compared with the control strains DE205B and DE205BΔ*upaB* (*P < 0.01). (**D**) The systemic infection experiment was conducted to assess the bacteria proliferation in duck lungs and blood. DE205BΔ*autA*, DE205BΔ*autR*, and DE205BΔ*autA/autR* enhanced the colonization in lungs and proliferation in the blood compared with the control strains DE205B and DE205BΔ*upaB*. A nonparametric Mann-Whitney U test was performed for statistical significance analysis (*P < 0.01). (**E**) Bactericidal assays revealed that the *autA* and *autR* mutants had higher resistance to non-infected duck serum compared to the control strains DE205B and DE205BΔ*upaB*. Bacteria were incubated with the normal duck serum for dilution (1:10 or 1:2) at 37 °C. Statistical significance analysis was performed using one-way ANOVA (*P < 0.01)

In order to determine whether AutA and AutR affected the adhesion of DE205B *in vitro*, the adhesion assay of *autA*/*autR* mutants and other variants was conducted by DF-1 cells. As shown in Fig. 7A, there was obvious reduction (69.3%/68.7% and 63.9%/61.5% for DE205BΔ*autA* and DE205BΔ*autR* at time points 2h/4h, respectively) of adherence in DF-1 cells compared to DE205B. The *autA* and *autR* mutants for upregulation of K1 capsule decreased its adhesion ability (*P* < 0.01) (Fig. 7A).

To test the intracellular survival and replication of DE205B variants in HD11 cells *in vitro*, intracellular survival assay was performed. The bacterial numbers in HD11 cells were determined at four time points (2 h, 4 h, 6 h, 8 h, and 16 h). As shown in Fig. 7B, *autA* and *autR* mutants enhanced its survival rates compared with the control strains DE205B and DE205BΔ*upaB in vitro*, and complemented strains for downregulation of K1 capsule decreased the ability of intracellular survival/replication within HD11 cells (*P* < 0.01) (Fig. 7B).

To determine the effect of *autA* and *autR* loss on APEC virulence for duck model, duck groups were challenged intratracheally with bacteria^25^,^29^. As shown in Fig. 7C, DE205BΔ*autA*, DE205BΔ*autR*, and DE205BΔ*autA/autR* enhanced the mortality rates and were ahead of duck death peak compared with DE205B and DE205BΔ*upaB* (*P* < 0.01). However, the survival rates of DE205BΔ*autA-*C*autA*, DE205BΔ*autR-*C*autR* were obviously higher than the mutant strains and close to the DE205B (*P* < 0.01) (Fig. 7C).

To measure the effect of *autA* and *autR* loss on APEC colonization *in vivo*, the systemic infection experiment was conducted to assess the bacteria proliferation in duck lungs and blood^25^,^29^. As shown in Fig. 7D, DE205BΔ*autA*, DE205BΔ*autR*, and DE205BΔ*autA/autR* enhanced the colonization in lungs compared with the control DE205B and DE205BΔ*upaB*, and bacterial proliferation in the blood among mutants showed a higher level of bacteremia than strains DE205B and DE205BΔ*upaB* (*P* < 0.01). DE205BΔ*autA-*C*autA* and DE205BΔ*autR-*C*autR* were obviously lower than the mutant strains but close to DE205B (*P* < 0.01) (Fig. 7D).

Due to higher proliferation in blood *in vivo* for sepsis, the serum resistance experiments were performed on all the strains. Bactericidal assays revealed that mutants for up-regulation expression of K1 capsule had higher resistance to non-infected duck serum compared with strains DE205B and DE205BΔ*upaB* (*P* < 0.01), and serum resistance of DE205BΔ*autA-*C*autA* and DE205BΔ*autR-*C*autR* were lower than the mutant strains and close to strains DE205B and DE205BΔ*upaB* (*P* < 0.01) (Fig. 7E).

### The novel APEC virulence regulatory networks

**Figure 8:**
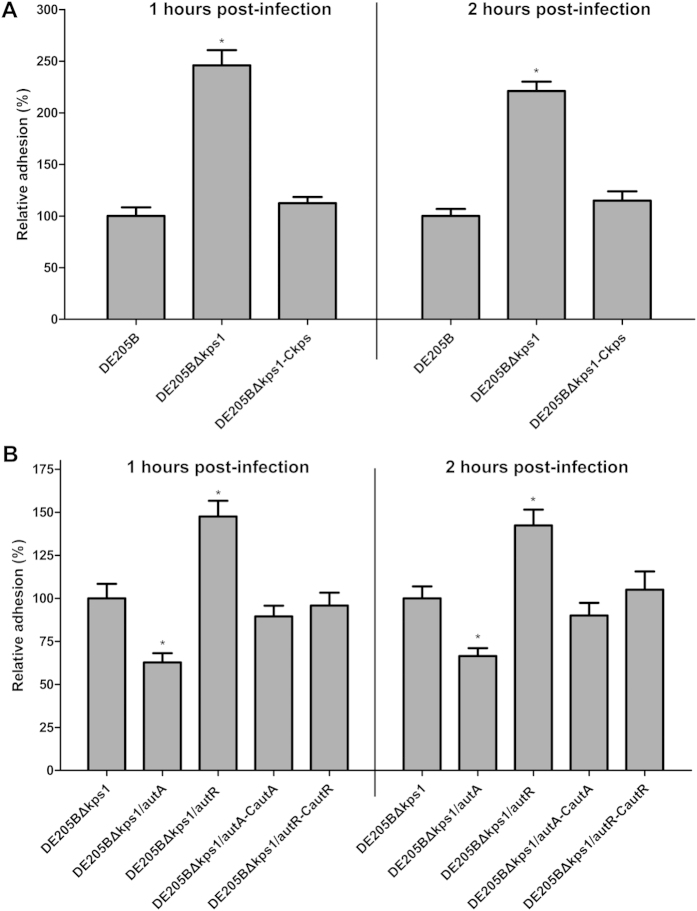
The effects of K1 capsule and AutA/AutR loss on DE205B adherence. (**A**) DE205BΔ*kps1* enhanced its adherence on DF-1 cells for 1h and 2h post-infection compared with DE205B. (**B**) DE205BΔ*kps1*/*autR* enhanced its adherence on DF-1 cells for 1 h and 2 h post-infection compared to DE205BΔ*kps1*, while adhesion capability of *autA* deletion in DE205BΔ*kps1* was impaired. Statistical significance analysis was performed using one-way ANOVA (*P < 0.01).

The loss of *autA* and *autR* result in phenotype effects on intracellular survival but not on adherence, suggesting that up-expressed K1 capsule in DE205B mutants might shield the virulence roles of AT adhesins UpaB and AatA. To test the effects of AutA and AutR loss on DE205B adherence, we constructed the *autA* and *autR* mutant variants on the basis of DE205BΔ*kps1*. DE205BΔ*kps1* increased adherence in DF-1 cells at 1 h and 2 h by approximately 2.46 and 2.21-fold compared to DE205B, respectively (*P* < 0.01), while the adhesion capability of complemented DE205BΔ*kps1*-C*kps* was restored to similar level of DE205B (*P* ≥ 0.05) (Fig. 8A). The adhesion capability of DE205BΔ*kps1*/*autR* were enhanced by approximately 47.6% and 42.4% at 1h and 2h compared with DE205BΔ*kps1*, while adhesion capability of *autA* deletion in DE205BΔ*kps1* was impaired by approximately 37.3% and 33.5% compared to DE205BΔ*kps1* (*P* < 0.01) (Fig. 8B). Moreover, adhesion capability of the complemented DE205BΔ*kps1*/*autA*-C*autA* and DE205BΔ*kps1*/*autR*-C*autR* were restored to original level of DE205BΔ*kps1* (*P* ≥ 0.05). Immunofluorescence assay was conducted to measure the adhesion abilities of DE205B variants for 2h infection, and the result was consistent with the results of plate counting (Figure S5). These results showed that *autA* and *autR* loss in DE205BΔ*kps1* could change the adhesion capability by co-regulating expression of UpaB and AatA.

We further conducted the RT-PCR analysis to confirm the heterogeneous expression pattern of K1 capsule and acid resistance systems regulated by AutA and AutR during DE205B infection *in vivo*. As shown in Figure S6, overexpression of AutA and AutR in complemented strains repressed the up-regulated expression of K1 capsule and down-regulated expression of acid resistance systems *in vivo*, and the expression trend of K1 capsule and acid resistance systems in complemented strains *in vivo* was opposite to DE205B during intracellular survival and serum resistance for sepsis *in vivo*. Therefore, based on the above transcription analysis and infection experiments, we suspected that the novel APEC virulence regulatory pathway: AutA and AutR in DE205B were responsible for co-regulation of AT adhesins, K1 capsule, and acid resistance systems under host-induced stimuli.

## Discussion

Bacteria can adopt a lifestyle switch to inhabit different host niches, which relies on multiple regulatory networks to cooperatively alter gene transcription^18^. APEC/ExPEC harbors several adhesins, including typical fimbriae and non-fimbrial autotranspoters (AT) adhesin, to adapt/colonize to extraintestinal specific niches^25^. Several transcriptional regulators control the expression of APEC/ExPEC adhesins. For example, the global regulator FNR alters UPEC virulence-associated phenotype by controlling expression of type I and P fimbriae, and TosR regulates the *in vivo* induced expression of nonfimbrial adhesin TosA^30^,^31^.

In this study, two novel regulators were identified and designated as AutA and AutR. AutA and AutR were predicted to have the C-terminal helix-turn-helix (HTH) motif similar to LuxR family regulator GerE, suggesting that the novel regulators could be classified as LuxR/GerE family regulators^19^,^20^,^22^. Furthermore, AutA and AutR harbored several putative enzyme domains for response signal receiver. Unlike two-component signaling systems (KguS/KguR and PhoQ/PhoP)^21^,^32^, there was no sensor kinase gene located in UpaB cluster. Our results showed that AutA and AutR might co-regulate the expression of AT adhesion UapB. It has been shown that virulence-related ATs, such as vacuolating autotransporter toxin (Vat) and AT UpaC, were also controlled by its adjacent transcriptional regulators^26^,^33^. However, unlike regulator TosR and adhesion TosA encoded by the an operon, genes (*autA, autR*, and *upaB*) in UapB cluster were not located in one operon, catering to the view that bacteria ATs are located in a single gene locus^34^. Apart from AutA and AutR, co-regulation (activator or repressor) on the target protein has been observed among other adjacent regulators, such as TosE/ TosF, gadX/gadW, and MarA/MarR^19^,^31^,^35^,^36^.

Many reports showed that transcriptional regulators, such as PafR, FNR, and ArcA, are not only alter the expression of its adjacent target protein, but also plays important roles in globally regulating gene transcription^21^,^30^,^37^,^38^. Transcriptome analysis among strains DE205B, DE205BΔ*autA*, and DE205BΔ*autR* showed that AutA and AutR coherently affected hundreds of genes expression. These regulated genes in mutants DE205BΔ*autA* and DE205BΔ*autR* exhibited similar trends, either up-regulation or down-regulation, compared with DE205B. Many different expressed genes were involved in APEC adhesion, capsule synthesis, and acid resistance, which were crucial for APEC infection and environmental adaptability. Therefore, AutA and AutR might act as global regulators and coherently regulate hundreds of genes expression in APEC.

The expression of APEC K1 capsule encoded by two operons (*kpsFEDUC* and *kpsMTDBACES*) was coherently regulated by the regulators AutA and AutR by transcriptome analysis. We constructed DE205B mutants and complemented strains to confirm their regulation on the expression of K1 capsule *in vitro*. King *et al*. showed the phenotypic heterogeneity in the expression of K1 capsule during UPEC strain UTI89 adaptive response, when UTI89 withstands changeable environment of the urinary tract and undertakes intracellular survival^17^. King *et al*. proposed that there might be unidentified regulators that can alter expression of K1 capsule under host-induced stimuli *in vivo*^17^. In this study, we confirmed that typical APEC strain DE205B also changed K1 capsule expression under different infection conditions. Moreover, DE205B K1 capsule was expressed in much higher level under intracellular survival within HD11 macrophages, and proliferation under duck sepsis than that of routine culture. Meanwhile, the transcription level of *autA* and *autR* for intracellular DE205B in DF-1/HD11 cells and isolated from infected duck blood was all decreasing compared with the control. Furthermore, overexpression of AutA and AutR in complemented strains repressed the expression of K1 capsule during DE205B intracellular survival within HD11 cells and proliferation in sepsis *in vivo*. These results agreed the hypothesis of King *et al*. and suggested that a heterogeneous expression pattern of K1 capsule during DE205B infection *in vivo* might be co-regulated by AutA and AutR under host-induced stimuli.

Since AutA and AutR co-regulating (activating or repressing) genes encoding virulence-related AT adhesins and K1 capsule, we further identified the roles of AutA and AutR in virulence-associated phenotype. The results of adhesion assay, intracellular survival assay, and bactericidal assays for DE205B variants showed that high expression of K1 capsule in DE205B mutants was associated with the increasing intracellular survival and serum resistance compared with DE205B. Our data showed that the adhesion capability of the unencapsulated DE205BΔ*kps1* was enhanced significantly compared to that of DE205B, and the adhesion capability of DE205BΔ*kps1*/*autR* was also enhanced compared to that of DE205BΔ*kps1*. Since the effects of *kpsFEDUC* deletion on adhesion capability, we suspected that the expression of K1 capsule could disrupt the AT adhesins roles in APEC adherence, catering to the notion that the unencapsulated APEC/ExPEC could enhance its binding and internalization rates than the encapsulated *E. coli*^15^,^16^,^17^. Furthermore, even shielding the virulence roles of short adhesins, virulence test for duck model confirmed that enhancing expression of K1 capsule in *autA* and *autR* mutants promoted adaptability during APEC early colonization and septicemia, and this phenomena was consistent with previous study for virulence roles of K1 capsule in APEC/ExPEC infection^12^,^13^,^14^,^15^. Combined with the heterogeneous expression pattern of AutA and AutR during DE205B infection, the novel APEC virulence regulatory pathway that AutA and AutR played the roles in APEC adaptive lifestyle switch by coordinately alter the expression of AT adhesins and K1 capsule^18^.

There was the other important finding that AutA and AutR co-activated the glutamate-dependent acid resistance (GDAR) and HdeABD acid resistance systems. Glutamate-dependent acid resistance is most efficient system for *E. coli* to survive in extremely acidic environments^39^,^40^. Unlike the higher prevalence of K1 capsule determinant and UpaB cluster in ExPEC B2 and D isolates, the acid fitness island is conserved distributed in almost the commensal *E. coli*, intestinal pathogenic *E. coli*, and ExPEC. The acid fitness island is a crucial adaptive evolution for *E. coli* to fitness in nature, including extremely acidic environments and pH varied host gut^27^,^39^,^40^. Transcriptome and RT-PCR analysis indicted that the transcription level of acid resistance-related genes for the DE205B under routine cultured condition (*in vitro*) were much higher that the *dnaE*. However, *autA* and *autR* mutants nearly lost its acid resistance properties and obviously decreased the transcription level of acid resistance-related genes. Previous reports have showed that the global regulators H-NS, CRP, RpoS repressed or activated the expression of gadABC and mdtEF multidrug efflux^41^,^42^,^43^. However, this study was the first confirmed that AutA and AutR co-regulated the acid-resistant encoding genes of AFI and its own regulators *gadE, gadW*, and *gadX*. With lower expression of AutA and AutR, the expression of acid resistance systems in AFI was regularly decreased during DE205B intracellular survival within HD11 macrophages and proliferation in sepsis. Since host extraintestinal specific niches maintaining near neutral pH environments, we speculated that DE205B might adopt an adaptive lifestyle switch to fitness the infection, and decreased excess expression of acid resistance systems compared to routine culture. Meanwhile, accompanied with higher expression of K1 capsule during DE205B infection, APEC utilized K1 capsule to undergo and suffer from oxidative stressors, serum complement components, and antimicrobial peptides^15^,^17^,^18^. Due to expression pattern of AT adhesins, K1 capsule, and acid resistance systems during DE205B infection *in vivo*, AutA and AutR undertook the roles for reciprocal phenotype regulation under host-induced stimuli^18^. Due to the changes in transcription of AutA/AutR during APEC intracellular survival between within DF-1 and HD-11 cells, there might be certain unknown factors (possibly APEC signaling kinases/regulators, small self-induced molecules or induced substance of host cells^19^,^20^) involved in regulating the AutA/AutR expression during APEC intracellular infection. And the unknown factors on APEC AutA/AutR expression might not present host-specific trait during APEC intracellular survival within DF-1 and HD-11 cell and in infected duck bloods.

This study indicated that AutA and AutR directly bound to the P_*upaB*_ promoter and acted as global regulators to co-regulate the transcription of hundreds of genes, suggesting that AutA and AutR could recognize the sequence-specific DNA binding sites for subsets of target promoters to exercise its activation or repression activity. Recent study has showed the LuxR can bind to specific promoter sequence, called the ideal LuxR consensus motif, by chromatin immunoprecipitation and nucleotide sequencing (ChIP-seq)^20^. The regulators GadEWX can recognize RpoS-binding sites to co-regulate the transcriptional regulatory networks of acid resistance systems in AFI^35^. In future studies to further elaborate the co-regulation mechanism, we will conduct experiments to explore the specific motif of AutA/AutR-binding sites.

## Experimental Procedures

### Ethics statement

All animal experimental protocols were handled according to the guidelines of Experimental Animal Management Measures of Jiangsu Province and were approved by the Laboratory Animal Monitoring Committee of Jiangsu Province, China.

### Strains and plasmids construction

The strains, plasmids, and the primers used in this study were described in Table S3. The high virulent DE205B (CVCC3991), its background (O2:K1; ST complex 95, ST140; ECOR B2; isolated from duck), acts as a typical model to unravel the APEC pathogenesis^4^,^25^. The DE205B mutants for the *autA, autR, lacI-Z*, and *kpsFEDUC* deletion were constructed by lambda red recombinase method previously described^44^. The primers for deletion were used to amplify antibiotic resistance cassettes from plasmid pKD4. The mutants for multiple genes deletion were constructed on basis of the red recombinase method, and the gene deletions were one by one conducted by the targeted antibiotic fragments recombination and eliminating antibiotic fragments^25^. The *upaB::lacZ-zeo* transcriptional reporter fusion in DE205BΔ*lacI-Z* were constructed on the basis of lambda red recombinase system^44^. The detailed operating steps referred to the Vigil *et al*.^45^. Briefly, the two targeted fragments were created (Figure S2): one was the *lacZ* product containing the regions of homology to 5′ end of *upaB* sequence (starting at the 30bp site after *upaB* start codon), and the other for kanamycin resistance cassettes was amplified from the pKD4 plasmid and contained the regions of homology to 3′ end of *upaB* sequence. Then fusion PCR was performed to connect the two fragments. Purified PCR products were recovered, and insert of fusion fragments in DE205BΔ*lacI-Z* chromosome was routinely conducted by the lambda red recombinase system in the pKD46 plasmid.

For the construction of complementary plasmids, a low-copy plasmid pGEN-MCS undertook the complementary carrier for the operon *kpsFEDUC* (containing its putative promoters)^46^,^47^. Due to long fragment connection, two pairs of primers were used to amplify the corresponding products with special restriction cutting sites. Then the digested PCR products were successively ligated into pGEN-MCS. For the construction of complementary plasmids for stable overexpression of AutA and AutR, a medium-copy plasmid pSTV28 (pACYC184 origin, TaKaRa) undertook the complementary carrier^25^,^48^. The *autA* and *autR* operons, including its predicted promoters, were amplified by the corresponding primers. Then the PCR products were digested and ligated into pSTV28. The plasmid products were electroporated into the corresponding DE205B mutants, and the complementary strains were cultured in routine condition^25^. The growth kinetics of all DE205B variants were performed as previous described^25^.The optical densities of three biological replicates in regular intervals were determined during growth in at 37 °C in liquid Luria Bertani (LB) medium.

To construct the plasmid overproducing MBP::AutA and MBP::AutR fusion proteins, the *autA* and *autR* genes were cloned into the expression plasmid pCold-malE, which was constructed by fusing *malE* coding sequence into the pCold I (TaKaRa) using single *NdeI* site. The *autA* and *autR* genes were amplified by the corresponding primers. The PCR products were digested and ligated into pCold-malE. The pCold-malE/autA and pCold-malE/autR were transformed into *E. coli* BL21 (DE3). The inducible expression of MBP::AutA and MBP::AutR proteins were performed using routine process^25^, and the purification of fusion proteins were conducted using a HisTrap high-performance column (GE Healthcare, Shanghai, China)^25^.

For more information and detailed descriptions on Experimental Procedures, see Text S2 in the supplemental material.

## Additional Information

**Accession codes**: The sequence of UapB cluster in APEC strain DE205B was submitted to GenBank, and the accession number is KT965673. RNA-Seq data are available at the NCBI BioProject database (http://www.ncbi.nlm.nih.gov/bioproject) under BioProject ID: PRJNA299423.

**How to cite this article**: Zhuge, X. *et al*. AutA and AutR, Two Novel Global Transcriptional Regulators, Facilitate Avian Pathogenic *Escherichia coli* Infection. *Sci. Rep.* **6**, 25085; doi: 10.1038/srep25085 (2016).

## Supplementary Material

Supplementary Information
