# Supplementary material for: AutA and AutR, Two Novel Global Transcriptional Regulators, Facilitate Avian Pathogenic Escherichia coli Infection
Source: Sci Rep. 2016 Apr 26;6:25085. doi: 10.1038/srep25085 (PMC4844996; doi:10.1038/srep25085)
Supplement: Supplementary Information [file srep25085-s1.doc]

**AutA and AutR, Two Novel Global Transcriptional Regulators,** **Facilitate Avian Pathogenic *Escherichia coli* Infection**

**Xiangkai Zhuge^1^, Fang Tang^1^, Hongfei Zhu^2^, Xiang Mao^3^, Shaohui Wang^3^, Zongfu Wu^1^, Chengping Lu^1^, Jianjun Dai^1^,*, Hongjie Fan^1^***

1, Key Lab of Animal Bacteriology, Ministry of Agriculture, Nanjing Agricultural University, Nanjing 210095, China

2 Beijing Veterinary Research Institute, Chinese Academy of Agricultural Sciences, Beijing 100193, China

3, Shanghai Veterinary Research Institute, Chinese Academy of Agricultural Sciences, Shanghai 200241, China

***Corresponding author:**

Jianjun Dai, Key Lab of Animal Bacteriology, Ministry of Agriculture, Nanjing Agricultural University, Nanjing 210095, China; Address: No.1 Weigang road, Nanjing, Jiangsu province, China; Phone: +86 25 84396566; Fax: +86 25 84432420; E-mail: daijianjun@njau.edu.cn

Hongjie Fan, Key Lab of Animal Bacteriology, Ministry of Agriculture, Nanjing Agricultural University, Nanjing 210095, China; Address: No.1 Weigang road, Nanjing, Jiangsu province, China; E-mail: fhj@njau.edu.cn

**Supplementary Text S1. The detailed descriptions on transcriptome analysis that regulators AutA and AutR coherently affected hundreds of genes expression**

To identify genes transcription (apart from *upaB*) regulated by AutA and AutR, genome-wide strand-specific transcriptome sequencing (RNA-seq) analysis was performed. The total RNA was isolated from three strains DE205B, DE205BΔ*autA*, and DE205BΔ*autR* under mid-logarithmic phase. The growth kinetics of DE205BΔ*autA* and DE205BΔ*autR* mutants showed the similar growth rates to DE205B (Figure S4), excluding the possibility that differential growth rate affected RNA-seq global expression profiles. The total RNA after removing rRNA was used to construct cDNA libraries and Hiseq4000 sequencing. In total, we obtained approximately 3.43Gbp to 3.68Gbp bases data for eight sequencing groups (each strain for two or three biological duplicates). The single-end reads were aligned to the APEC IMT5155 genome sequence using TopHat program. Of note, DE205B and IMT5155 share closest relationship and belong to ST95 and serotype O2:K1 APEC . Our unpublished DE205B draft genome sequence showed above 99.99% similarity with gene contents and loci of IMT5155.

The cDNA reads of DE205B and mutants for genome-wide landscape were adjusted to same scale, mapped to and plotted as log_10_^FPKM+1^ values over the IMT5155 genome (Figure 4A). The RNA-seq analysis showed that mutants DE205BΔ*autA* and DE205BΔ*autR* shared similar genomic expression profiles except for UpaB expression (Figure 4A). However, transcription levels of 223 genes, containing 41 operons, in DE205BΔ*autA* and DE205BΔ*autR* exhibited changes (> 2-fold, *p* value < 0.05) compared with DE205B. The detailed information for 223 genes was listed in Table S1, which showed the genes were either up-regulated or down-regulated in DE205BΔ*autA* and DE205BΔ*autR* (Table S1). Fifty genes that differently expressed were plotted in the global expression map (Figure 4A). To validate the RNA-seq analysis, RT-PCR of these 50 genes was conducted among mutants and wildtype DE205B. The quantitative PCR result (Table S2) showed the similar tendency to RNA-seq analysis. Based on the RNA-seq analysis, AutA and AutR might coherently regulate hundreds of genes expression.

There were 67 genes down-regulated in DE205BΔ*autA* and DE205BΔ*autR* compared with DE205B, included several metabolism-related genes, such as *talA* and *tktB* encoding transaldolases for pentose phosphate pathway, *glpX* and *glpFK* operon encoding the glycerol facilitator to facilitate glycerol across the cytoplasmic membrane, and hydrogenase-1 operon (*hyaABCDEF*) encoding Fe-S cluster-containing hydrogenases in H_2_ oxidation under *E. coli* anaerobic conditions (Table S1). It was worth mentioned that the *aatA* encoding APEC autotransporter adhesin, which is an important virulence factor for APEC pathogenicity[^3^](#_ENREF_3), was down-regulated 8.7-fold for DE205BΔ*autA* and 8.2-fold for DE205BΔ*autR* relative to DE205B (*P*<0.01). Except for the *gadBC* operon, the most significantly down-regulated (near 2^7^-fold) genes focused on the acid fitness island (AFI), which undertakes critical roles in *E. coli* acid resistance through several pathways [^4^](#_ENREF_4) (*P*<0.01) (Table S1). The genetic context of AFI was described in Figure 4A and contained five operons, including *slp-yhiF*, *hdeAB-yhiD*, *gadE-mtdEF*, *gadXW* and *gadAX* . Additionally, a gene cluster encoding the heme transport system is inserted in AFI of several ExPEC and *Enterohemorrhagic* *Escherichia coli* (EHEC). The *gadA* in AFI and *gadBC* operon encodes GadA/GadB decarboxylases and GadC membrane transporter protein for glutamate, which is called glutamate-dependent acid resistance system (GDAR) and acts as the most effective system for acid resistance [^6^](#_ENREF_6). The two periplasmic chaperones HdeA and HdeB encoded by *hdeAB* and acid-resistance membrane protein HdeD is the another acid resistance system in AFI to protect *E. coli* from extremely acidic conditions[^7^](#_ENREF_7). The GadEWX-transcriptional regulatory networks encoded by the significantly upregulated genes *gadE*, *gadW*, and *gadX*  in mutants of *autA* or *autR* deletion coherently regulates the expression of acid resistance systems in AFI [^8^](#_ENREF_8).

There were 156 genes up-regulated in DE205BΔ*autA* and DE205BΔ*autR* compared to DE205B, including many operons encoding metabolic-related pathways, such as *dsdCXA* for D-serine transporter system, and oligopeptide permease ABC transport operon (Table S1). Unlike down-regulated hydrogenase-1 operon (*hyaABCDEF*), the *hybOABCDE* operon for hydrogenases 2 was up-regulated more than 4-fold (*P*<0.01). Of note, the expression of universal stress proteins (uspA, uspD, and uspF) were up-regulated in *autA* and *autR* deletion mutants, and cold shock-like protein CspD was up-regulated in DE205BΔ*autA* and DE205BΔ*autR* compared to the wildtype DE205B. The cold shock protein CspA was significantly down-regulated (near 19.7-fold) in *autA* and *autR* deletion mutants (*P*<0.01).

Most importantly, the two operons (*kpsFEDUC* and *kpsMTDBACES*) of K1 capsule determinant encoding synthesis and transport proteins of *E. coli* K1 polysialic acid capsule were obviously up-regulated in *autA* and *autR* deletion mutants [^9^](#_ENREF_9) (Table S1; Figure 4A). It was important to suggest that regulators AutA and AutR might coherently regulate the key virulence factor K1 capsule biosynthesis. Thus, AutA and AutR might facilitate several APEC infection routes, such as antiphagocytosis, intracellular macrophage survival, and avian blood/serum resistance [^10-13^](#_ENREF_10). Moreover, there was the up-expression of outer membrane protein TraT for serum resistance, OmpT, and OmpW in *autA* and *autR* mutants. The OmpT could degrade the antimicrobial peptide LL-37 and promote *E. coli* extraintestinal infection and intracellular survival , and OmpW also increased *E. coli* survival during phagocytosis[^16^](#_ENREF_16). Besides K1 antigen, serotype O2-antigen genes, containing *wzx* (encoding O-antigen flippase) and *wzy* (encoding O-antigen polymerase), were also up-regulated in *autA* and *autR* deletion mutants (Table S1).

Our data showed that 22 regulators for the feedback of *autA* or *autR* deletion were differential expressed, such as up-regulated HdfR and biofilm regulator BssR (Table S1). The regulator HdfR represses Gad acid resistance pathway and flhDC master operon of flagellar motility, and BssR increases biofilm formation ability and inversely controls flagella motility . The transcriptome analysis for differential expressed regulators suggested that AutA and AutR might participate in regulatory cascade networks for APEC adaptive lifestyle.

**References**

1. Zhu Ge, X. *et al.* Comparative genomic analysis shows that avian pathogenic *Escherichia coli* isolate IMT5155 (O2:K1:H5; ST complex 95, ST140) shares close relationship with ST95 APEC O1:K1 and human ExPEC O18:K1 strains. *PLoS One* **9**, e112048 (2014).

2. Ewers, C. *et al.* Avian pathogenic, uropathogenic, and newborn meningitis-causing *Escherichia coli*: how closely related are they? *Int J Med Microbiol* **297**, 163-76 (2007).

3. Zhu-Ge, X.K. *et al.* The effects of upaB deletion and the double/triple deletion of *upaB*, *aatA*, and *aatB* genes on pathogenicity of avian pathogenic *Escherichia coli*. *Appl Microbiol Biotechnol* **99**, 10639–10654 (2015).

4. Tramonti, A., De Canio, M. & De Biase, D. GadX/GadW-dependent regulation of the *Escherichia coli* acid fitness island: transcriptional control at the gadY-gadW divergent promoters and identification of four novel 42 bp GadX/GadW-specific binding sites. *Mol Microbiol* **70**, 965-82 (2008).

5. Tree, J.J. *et al.* Transcriptional regulators of the GAD acid stress island are carried by effector protein-encoding prophages and indirectly control type III secretion in enterohemorrhagic *Escherichia coli* O157:H7. *Mol Microbiol* **80**, 1349-65 (2011).

6. Castanie-Cornet, M.P. *et al.* Acid stress response in *Escherichia coli*: mechanism of regulation of gadA transcription by RcsB and GadE. *Nucleic Acids Res* **38**, 3546-54 (2010).

7. Hong, W., Wu, Y.E., Fu, X. & Chang, Z. Chaperone-dependent mechanisms for acid resistance in enteric bacteria. *Trends Microbiol* **20**, 328-35 (2012).

8. Seo, S.W., Kim, D., O'Brien, E.J., Szubin, R. & Palsson, B.O. Decoding genome-wide GadEWX-transcriptional regulatory networks reveals multifaceted cellular responses to acid stress in *Escherichia coli*. *Nat Commun* **6**, 7970 (2015).

9. Steenbergen, S.M. & Vimr, E.R. Biosynthesis of the *Escherichia coli* K1 group 2 polysialic acid capsule occurs within a protected cytoplasmic compartment. *Mol Microbiol* **68**, 1252-67 (2008).

10. Mellata, M. *et al.* Role of avian pathogenic *Escherichia coli* virulence factors in bacterial interaction with chicken heterophils and macrophages. *Infect Immun* **71**, 494-503 (2003).

11. Mellata, M. *et al.* Role of virulence factors in resistance of avian pathogenic *Escherichia coli* to serum and in pathogenicity. *Infect Immun* **71**, 536-40 (2003).

12. Anderson, G.G., Goller, C.C., Justice, S., Hultgren, S.J. & Seed, P.C. Polysaccharide capsule and sialic acid-mediated regulation promote biofilm-like intracellular bacterial communities during cystitis. *Infect Immun* **78**, 963-75 (2010).

13. Kim, K.J., Elliott, S.J., Di Cello, F., Stins, M.F. & Kim, K.S. The K1 capsule modulates trafficking of *E. coli*-containing vacuoles and enhances intracellular bacterial survival in human brain microvascular endothelial cells. *Cell Microbiol* **5**, 245-52 (2003).

14. He, X.L. *et al.* Role of uropathogenic *Escherichia coli* outer membrane protein T in pathogenesis of urinary tract infection. *Pathog Dis* **73**(2015).

15. Brannon, J.R., Thomassin, J.L., Desloges, I., Gruenheid, S. & Le Moual, H. Role of uropathogenic *Escherichia coli* OmpT in the resistance against human cathelicidin LL-37. *FEMS Microbiol Lett* **345**, 64-71 (2013).

16. Wu, X.B. *et al.* Outer membrane protein OmpW of *Escherichia coli* is required for resistance to phagocytosis. *Res Microbiol* **164**, 848-55 (2013).

17. Krin, E., Danchin, A. & Soutourina, O. Decrypting the H-NS-dependent regulatory cascade of acid stress resistance in *Escherichia coli*. *BMC Microbiol* **10**, 273 (2010).

18. Domka, J., Lee, J. & Wood, T.K. YliH (BssR) and YceP (BssS) regulate *Escherichia coli* K-12 biofilm formation by influencing cell signaling. *Appl Environ Microbiol* **72**, 2449-59 (2006).

**Supplementary Text S2. The more information and detailed descriptions on Experimental Procedures**

**Ethics statement**

All animal experimental protocols were handled according to the guidelines of Experimental Animal Management Measures of Jiangsu Province and were approved by the Laboratory Animal Monitoring Committee of Jiangsu Province, China.

**Strains and plasmids construction**

The strains, plasmids, and the primers used in this study were described in Table S3. The high virulent DE205B (CVCC3991), its background (O2:K1; ST complex 95, ST140; ECOR B2; isolated from duck), acts as a typical model to unravel the APEC pathogenesis . The DE205B mutants for the *autA*, *autR*, *lacI-Z*, and *kpsFEDUC* deletion were constructed by lambda red recombinase method previously described [^3^](#_ENREF_3). The primers for deletion were used to amplify antibiotic resistance cassettes from plasmid pKD4. The mutants for multiple genes deletion were constructed on basis of the red recombinase method, and the gene deletions were one by one conducted by the targeted antibiotic fragments recombination and eliminating antibiotic fragments[^2^](#_ENREF_2). The *upaB::lacZ-zeo* transcriptional reporter fusion in DE205BΔ*lacI-Z* were constructed on the basis of lambda red recombinase system[^3^](#_ENREF_3). The detailed operating steps referred to the Vigil et al.[^4^](#_ENREF_4). Briefly, the two targeted fragments were created (Figure S2): one was the *lacZ* product containing the regions of homology to 5’ end of *upaB* sequence (starting at the 30bp site after *upaB* start codon), and the other for kanamycin resistance cassettes was amplified from the pKD4 plasmid and contained the regions of homology to 3’ end of *upaB* sequence. Then fusion PCR was performed to connect the two fragments. Purified PCR products were recovered, and insert of fusion fragments in DE205BΔ*lacI-Z* chromosome was routinely conducted by the lambda red recombinase system in the pKD46 plasmid.

For the construction of complementary plasmids, a low-copy plasmid pGEN-MCS undertook the complementary carrier for the operon *kpsFEDUC* (containing its putative promoters). Due to long fragment connection, two pairs of primers were used to amplify the corresponding products with special restriction cutting sites. Then the digested PCR products were successively ligated into pGEN-MCS. For the construction of complementary plasmids for stable overexpression of AutA and AutR, a medium-copy plasmid pSTV28 (pACYC184 origin, TaKaRa) undertook the complementary carrier . The *autA* and *autR* operons, including its predicted promoters, were amplified by the corresponding primers. Then the PCR products were digested and ligated into pSTV28. The plasmid products were electroporated into the corresponding DE205B mutants, and the complementary strains were cultured in routine condition[^2^](#_ENREF_2). The growth kinetics of all DE205B variants were performed as previous described[^2^](#_ENREF_2).The optical densities of three biological replicates in regular intervals were determined during growth in at 37°C in liquid Luria Bertani (LB) medium.

To construct the plasmid overproducing MBP::AutA and MBP::AutR fusion proteins, the *autA* and *autR* genes were cloned into the expression plasmid pCold-*malE*, which was constructed by fusing *malE* coding sequence into the pColdⅠ(TaKaRa) using single *NdeI* site. The *autA* and *autR* genes were amplified by the corresponding primers. The PCR products were digested and ligated into pCold-*malE*. The pCold-*malE*/*autA* and pCold-*malE*/*autR* were transformed into *E. coli* BL21 (DE3). The inducible expression of MBP::AutA and MBP::AutR proteins were performed using routine process[^2^](#_ENREF_2), and the purification of fusion proteins were conducted using a HisTrap high-performance column (GE Healthcare, Shanghai, China)[^2^](#_ENREF_2).

**DNA and protein sequence analysis**

Two online prediction tools at (<http://linux1.softberry.com/berry.phtml?topic=bprom&group=programs&subgroup=gfindb>) and (<http://www.fruitfly.org/seq_tools/promoter.html>) were used to predict the transcription promoter of *autA* and *autR* genes. The bioinformatic analysis for protein structure and the homology domain prediction were performed by BLASTP on NCBI website, HHpred program, online MOTIF Search, and Phyre^2^ server server under the normal parameters and database.

**PCR genotyping**

The APEC strain collection (n=347) was used to determine the presence of UpaB cluster . The primers corresponding to four ORFs of UpaB cluster were designed with the conserved sequence, the similar annealing temperature, and the clear distinction for the size of the amplified fragments. Multiplex PCR was performed according to Johnson et al.[^9^](#_ENREF_9).

**RNA isolation, rRNA removing, and RNA sequencing**

The total RNA isolated from three strains DE205B, DE205BΔ*autA*, and DE205BΔ*autR*, which were cultured in LB (pH 7.4) under mid-logarithmic phase, was extracted using TRIzol® Reagent (Invitrogen) with DNase I (TaKara) to remove the genomic DNA according to the manufacturer’s instruction. Then RNA concentration and quality was determined using 2100 Bioanalyser (Agilent) and quantified using the ND-2000 (NanoDrop Technologies). High-quality RNA samples (OD260/280=1.8~2.2, OD260/230≥2.0, RIN≥6.5, 28S:18S≥1.0, >10μg) were stored at −80°C until use to construct sequencing library. The initial 10 μg total RNA was used to construct RNA sequencing library. First, Rrna was removed according to the manual of MICROBExpress™ Bacterial mRNA Enrichment Kit (Life Technologies, Carlsbad, CA). The remained rRNA-free RNA was used to construct one RNA-seq library by TruSeq^TM^ RNA sample preparation Kit (Illumina, San Diego, CA). Two or three biological duplicates were introduced for each strain. cDNA synthesis, end repair, A-base addition and ligation of the Illumina-indexed adaptors were conducted according to the Illumina’s guidelines of the manufacturers. Multiplex libraries were performed using barcoded primers, and the size of cDNA target fragments for the purified libraries were selected of 200–300 bp. The size distribution of paired-end libraries was analyzed using a Bioanalyzer and quantified by quantitative RT-PCR using a Kapa library quantification kit (Kapa Biosystems, Boston, MA). After pooled accordingly, the libraries were sequenced on an Illumina HiSeq 2500 platform with 125-bp pair end sequencing model according to the Illumina protocols.

**Data analyses**

SeqPrep (<https://github.com/jstjohn/SeqPrep>) and Sickle (<https://github.com/najoshi/sickle>) with default parameters were used to trim and control the quality of the raw paired end reads for RNA sequencing data. Due to the genome of DE205B was not completely sequenced, the complete genome sequences of APEC O2:K1 strain IMT5155 which shares closest evolutionary relationship (belonging to ST95 and serotype O2:K1 APEC) and similarity for more than 99.99% with the gene contents of DE205B draft genome (unpublished data for DE205B draft genome), acted as the reference genome to map the RNA sequencing data [^1^](#_ENREF_1). The clean reads were aligned to IMT5155 genome using the Bowtie2, which was a useful program to identify the gene expression for RNA-Seq data[^10^](#_ENREF_10). To determine the DEGs (differential expression genes) between the two different samples, the transcriptional level for each annotated genes was calculated using the fragments per kilobase of exon per million mapped reads (FPKM) method . Cuffdiff (<http://cufflinks.cbcb.umd.edu/>)[^13^](#_ENREF_13) was performed to assess the differential expression genes with more than five FPKM values. The DEGs between two samples need to meet the selected criteria: i) the logarithmic of fold change was greater than 2 and the Bonferroni-corrected *P*-value should be less than 0.05. Heat map for bioinformatics statistics was drawn to fully understand the differentially expressed genes for genome-wide landscape.

**Reverse transcription PCR and quantitative real-time RT-PCR**

The total RNA was extracted from bacteria cultured in LB and isolated from blood for infected duck [^2^](#_ENREF_2) using an E.Z.N.A. bacterial RNA kit (Omega Bio-Tek, Beijing, China) according to the manufacturer’s protocol. The total RNA was treated with DNase for one hour, and then PCR was conducted by the templates for RNA samples without reverse transcription to confirm free from contaminating DNA for the treated RNA samples. For the total RNA extracted from the infected cells during bacteria infecting DF-1/HD11 cells, the monolayer cells were incubated with bacteria at an infection ratio 1:200 with different time intervals or additional antibiotic treatment, and then the cells were washed with PBS for three times, and TRIZOL (invitrogen) was added to wells to lyse cells and extract the total RNA from the infected cells. The RNA samples were treated with DNase as the described above. In order to enrich the concentration of bacteria mRNA, the total RNA from the infected cells was treated with MICROB*Enrich*^TM^ Kit (Ambion; catalog no. AM1901) to remove host RNA , and then processed with MICROB*Express*^TM^ (Ambion; catalog no. AM1905) to deplete the bacterial rRNA. The each sample was repeated the above processes with three biological replicates.

For the co-transcription test, the treated bacteria RNA was reverse transcribed in cDNA using a SuperScript II reverse transcriptase kit (Invitrogen). The qPCR by primers that spanned the 3’ end of one gene to the 5’ end of the adjacent genes (Table S3) was performed to assess the co-transcription of intergenic regions as previously described . Reactions without reverse transcriptase acted as a negative control to detect the DNA contamination.

The real-time PCR was conducted as previously described [^2^](#_ENREF_2), and the primers for qRT-PCR were shown in Table S3. The transcription level of the housekeeping gene *dnaE* acted as a reference to determine the expression level of targeted genes with the ΔΔ*C_T_* method as previously described . Quantitative real-time RT-PCR (qRT-PCR) was conducted with the AceQ qPCR SYBR Green Master Mix (Vazyme, Nanjing) according to the manufacture’s instruction[^2^](#_ENREF_2).

**Electrophoretic mobility shift assays**

To determine the binding of AutA and AutR to DNA probe of *upaB* promoter, electropho-retic mobility shift assays (EMSAs) were conducted using the commercialized EMSA kit (Invitrogen, California) according to the manufacturer’s protocol[^15^](#_ENREF_15).

MBP::AutA and MBP::AutA fusion proteins were successfully expressed and purified in *E. coli* as the described above. For the DNA probes, the predicted P*_upaB_* DNA fragment, (200bp in size, starting from upstream position -170bp to downstream position +30bp relative to the position of the translational start codon) and the negative control DNA fragment (200bp in size for *upaB* coding region) were PCR amplified with the corresponding primers (Table S3), and the products were purified using an agarose gel DNA fragment recovery kit (TaKaRa). EMSAs were conducted by adding increasing amounts of MBP::AutA and MBP::AutA fusion proteins (0 to 200 ng) to the DNA probe (50 ng) in premixed binding buffer of EMSA kit. The reactions were carried out for 45 min at room temperature, and the samples were injected in the 6% polyacrylamide gels, and the electrophoresis was performed in 0.5×TBE buffer at 200 V for 30 min. The gels were photographed using the gel imaging system (Bio-Rad) after incubation with 1×SYBR Gold nucleic acid staining solution in 0.5×TBE buffer for 30 min.

***In vitro* transcription assays**

To determine the effect of AutA and AutR on *upaB* transcription, the *in vitro* transcription assays were preformed according to the Uppal et al. described[^17^](#_ENREF_17). A P*_upaB_*-*upaB* DNA fragment (starting from upstream position -170bp to downstream position +400bp relative to the position of the translational start codon) and a control P*_tac_*-*malE* DNA (containing P*_tac_* and partial *malE* fragment from plasmid pMAL-c2x) were PCR amplified with the corresponding primers (Table S3), and the products were purified using an agarose gel DNA fragment recovery kit (TaKaRa). The *in vitro* transcription assays were conducted using the commercial *E. coli* RNA polymerase holoenzyme (USB) with or without added MBP::AutA and MBP::AutR in the reaction. Briefly, reactions (20µL) were carried out with the above DNA fragments in a buffer containing 100 mM KCl, 20 mM Tris-HCl (pH 8.0), 3 mM MgCl2, 1 mM DTT, 100 µg of bovine serum albumin/ml, and 200 µM cAMP. The reaction mixture with or without MBP::AutA and MBP::AutR (10 ng, respectively) was incubated with 1U of *E. coli* RNA polymerase holoenzyme at 37 °C for 15 min, followed with adding dNTP (deoxy-ribonucleoside triphosphate, 200 µM) and further reaction at 37°C for 15 min. The reaction products were treated with DNase I (Vazyme, Nanjing) to remove the DNA templates. The RNA synthesized *in vitro* was reverse transcribed in cDNA using a SuperScript II reverse transcriptase kit (Invitrogen). The real-time PCR was conducted using the AceQ qPCR SYBR Green Master Mix (Vazyme, Nanjing) as previously described [^2^](#_ENREF_2), and the primers for qRT-PCR were shown in Table S3. The transcription level of the corresponding RNA synthesized *in vitro* was determined with the ΔΔ*C_T_* method as previously described . The qRT-PCR products were run on a 2% agarose gel, and then gels were photographed using the gel imaging system (Bio-Rad) to verify transcription difference. The *In vitro* transcription assays were repeated three times.

**β-Galactosidase assays**

The bacteria harboring the *upaB*::*lacZ* transcriptional fusions described above were cultured overnight in LB medium. The overnight cultured bacteria were diluted 1:100 in LB medium and grown to mid-log phase (OD_600_ 0.6 to 0.8) at 37 °C. Then after placing on ice and centrifugation, the bacteria were resuspended and diluled 1:10 in Z buffer. The Miller assay for β-galactosidase activity was conducted using *ortho*-nitrophenyl-β-galactoside (ONPG) as the substrate as previously described . The tests for β-Galactosidase activity were repeated three times.

**ELISA**

The ELISA to detect the K1 capsule antigen among DE205B variants was performed according to the Schneider et al. described [^18^](#_ENREF_18). Briefly, the 96-well plates were overnight coated at 4℃ with 0.2 ml bacteria (10^9^ CFU /well). After washed with PBS containing 0.5% Tween 20 (washing buffer), the plates were blocked with PBS plus 2% bovine serum albumin (Sigma) for 1h at 37 °C. Then rabbit anti-meningococcus type B antiserum (Neisseria Meningitidis Antiserum Gp B, BD Difco™), which also raised against *E. coli* K1 polysaccharide capsules due to antigenical identity between meningococcal group B and *E. coli* K1 polysaccharide capsule , was diluted 1:100 in PBS plus 0.5% BSA and added to ELISA plate wells. After incubation for 90 min at 37 °C, the plates were washed with PBS and then incubated with horseradish peroxidase (HRP)-conjugated anti-rabbit IgG (Vazyme, Nanjing). The optical density was measured at 490 nm, and the ELISA assays were repeated three times.

**Capsule staining**

The K1 capsule of *E. coli* K1 strains was observed with negative staining using Capsule Staining kit (Hopebio, Qingdao). The bacterial cells grown to mid-log phase in LB were washed twice, and the bacteria were resuspended in PBS. The Bacterial cells (10 µL) were spotted on microscope slides, and the fixation of cells was performed with air dry without heat. Then cells were incubated with Solution A containing 1% crystal violet for 5 min, and washed twice with Solution B containing 20% copper sulfate. After drying with absorbent paper, the slides of stained cells were added with cedar oil and conducted the microscopic observation. The color of stained bacteria was purple with pale purple of background color, and capsule of bacteria was colorless.

**Acid resistance assays**

Acid resistance assays were performed according to the procedures of previous study with some modifications . The bacterial cells grown to mid-log phase in LB (pH 7.4) were harvested by centrifugation and resuspended in LB (pH 7.4) to 1.0×10^9^ CFU/ml. The bacterial cells were diluted 1:10 into LB medium at pH 2.0, adjusted to pH 2.0 with concentrated HCl. Then the pH 2.0 treated cells for acid challenge were inoculated at 37 °C without shaking for 2h. The pH 7.4 treated cells, inoculated at 37 °C without shaking for 2h, was the negative control. The cells samples were serially diluted and plated on LB agar plates (pH 7.4) for counting. Acid resistance assays were carried out in triplicate. Percent survival for bacterial acid resistance was measured as follows [^22^](#_ENREF_22): ((CFU per ml at time 2 h)/(CFU per ml at time zero)) ×100. In parallel, bacterial cells exposed to acid challenge were serially diluted 1:10 in LB (pH 7.4), and the 10 µL cells for each dilution were spotted onto LB agar plates (pH 7.4).

**Serum bactericidal test**

Bactericidal assays were conducted as the described previously with some modifications[^7^](#_ENREF_7). The bacterial grown to mid-log phase in LB were centrifuged and washed with PBS, and the bacterial cells were resuspended in PBS to 1.0×10^8^ CFU/ml. The bacterial cells were then diluted 1:10 or 1:2 into the normal duck serum, which were obtained from ten-day-old HBK-Q-SPF ducks and had no APEC antibodies, detected using ELISA with DE205B lysate antigen. After incubation at 37 °C without shaking for 1h, the cells samples were serially diluted and plated on LB agar plates for counting. Serum bactericidal tests were carried out in triplicate. Percent survival for bacterial serum resistance were measured among DE205B variants relative to the wildtype DE205B as following ((CFU per ml at time 1 h for DE205B variants)/(CFU per ml at time 1 h for the wildtype DE205B)) ×100.

**Cells infection assays**

The adhesion and invasion assays were performed according to the previous described . Briefly, the monolayer DF-1 cells were infected with DE205B variants at a multiplicity of infection of 100 at several time points. Then the infected cells were washed with PBS, and lysed using the 0.1% Triton X-100. The plate counting for the total number of adherent bacteria was carried out with the serially diluted cell suspension. For the number of invasive bacteria, the infected cell were washed with PBS, and then treated with DMEM containing gentamicin (100μg/mL) for 1h to kill the adherent bacteria. The plate counting was conducted to determine the number of invasive bacterial. These experiments were performed in triplicate.

The intracellular survival assays were performed according to the previous described[^7^](#_ENREF_7). The monolayer macrophage HD-11 cells were infected with DE205B variants at a multiplicity of infection of 50 at several time points. After 1h of infection, infected cells were treated with gentamicin for 1h to kill extracellular bacteria. After 1h of treatment with gentamicin, internalized bacteria for 2h of infection acted as the initial number of intracellular bacteria with plate counting as the described above. After 1h of infection, the bacteria of intracellular survival within HD11 cells treated with gentamicin for the different time points (3h, 5h, 7h, and 15h) were measured with plate counting. Intracellular survival was calculated as change (n-fold) in bacterial number at a given time point relative to initially internalized bacteria. The intracellular survival assays were performed in triplicate.

**Immunofluorescent imaging assays**

For the imaging of the bacteria capsule during its intracellular infection, the immunofluorescent imaging assays were conducted according to the King et al. described with some modifications[^19^](#_ENREF_19). Briefly, the infected cells were washed with PBS and fixed in 3% paraformaldehyde, and then treated with 0.1% Triton X-100 in PBS for 3 min. After blocked with PBS plus 5% BSA, the capsule was then stained using the rabbit anti-meningococcus type B antiserum (Neisseria Meningitidis Antiserum Gp B, BD Difco™) at 37 °C for 2h. The infected cells were washed with PBS three times and treated with FITC goat anti-rabbit IgG (EarthOx, San Francisco) at 37°C for 1h. The The DF-1 cells were incubated with DAPI and Phalloidin (actin staining; TRITC conjugated) at 37°C for 40 min. Samples were washed three times and conducted to immunofluorescent imaging using a Zeiss LSM-510 META confocal laser scanning microscope. The fluorescence (Green) of K1 capsule were observed, and extracellular and intracellular bacteria stained with DAPI and K1 antibody were differentiated with cytoskeleton staining (Red).

For the imaging of the bacteria counting in infected DF-1 cells, the infected cells were treated as the described above, and then incubated with a polyclonal rabbit anti-DE205B serum, which were prepared in the previous study . Then the infected cells were treated with FITC goat anti-rabbit IgG (EarthOx, San Francisco), DAPI, and Phalloidin (actin staining; TRITC conjugated) as the described above. The immunofluorescent imaging of bacteria was detected using a Zeiss LSM-510 META confocal laser scanning microscope. The number of bacteria adhering to 50 DF-1 cells could be directly counted from immunofluorescent imaging. The mean ± standard deviation number of adherent bacteria per DF-1 cells was shown for each strain.

**Animal experiments**

The seven-day-old HBK-Q-SPF ducks for duck model were used to determine the effect of *autA* and *autR* loss on APEC virulence as the previously described . The duck groups (20 ducks for each group) were challenged intratracheally with bacteria at 5.0×10^5^ CFU/duck (dose/duck similar to LD_50_ of wild-type DE205B) . The mortality was calculated at the 7th day after postinfection, and the survival/mortality rates of eight groups were assessed.

To measure the effect of *autA* and *autR* loss on APEC colonization *in vivo*, the systemic infection experiment of duck model was conducted to assess the bacteria proliferation in duck lungs and blood . The duck groups were challenged intratracheally with bacteria at 2.0×10^6^ CFU/duck (dose/duck similar to LD_90_ of wild-type DE205B) . 10 ducks for each group were euthanized and dissected at 24h post-infection to conduct systemic infection experiment. The number of bacteria colonizing in lungs and level of bacteremia in the blood brains were determined as follows : organ samples and blood were obtained from infected ducks. The samples were weighed, suspended in PBS (1 ml/g), and homogenized; the blood and homogenized tissues were serially diluted and plated on LB agar plates for counting.

**Accession numbers**

The sequence of UapB cluster in APEC strain DE205B was submitted to GenBank, and the accession number is KT965673. RNA-Seq data are available at the BioProject database (http://www.ncbi.nlm.nih.gov/bioproject) under BioProject ID: PRJNA299423.

**References**

1. Zhu Ge, X. *et al.* Comparative genomic analysis shows that avian pathogenic *Escherichia coli* isolate IMT5155 (O2:K1:H5; ST complex 95, ST140) shares close relationship with ST95 APEC O1:K1 and human ExPEC O18:K1 strains. *PLoS One* **9**, e112048 (2014).

2. Zhu-Ge, X.K. *et al.* The effects of *upaB* deletion and the double/triple deletion of *upaB*, *aatA*, and *aatB* genes on pathogenicity of avian pathogenic *Escherichia coli*. *Appl Microbiol Biotechnol* **99**, 10639–10654 (2015).

3. Datsenko, K.A. & Wanner, B.L. One-step inactivation of chromosomal genes in *Escherichia coli* K-12 using PCR products. *Proc Natl Acad Sci U S A* **97**, 6640-5 (2000).

4. Vigil, P.D. *et al.* The repeat-in-toxin family member TosA mediates adherence of uropathogenic *Escherichia coli* and survival during bacteremia. *Infect Immun* **80**, 493-505 (2012).

5. Lane, M.C., Alteri, C.J., Smith, S.N. & Mobley, H.L. Expression of flagella is coincident with uropathogenic *Escherichia coli* ascension to the upper urinary tract. *Proc Natl Acad Sci U S A* **104**, 16669-74 (2007).

6. Spurbeck, R.R. *et al.* Fimbrial profiles predict virulence of uropathogenic *Escherichia coli* strains: contribution of ygi and yad fimbriae. *Infect Immun* **79**, 4753-63 (2011).

7. Wang, S. *et al.* DotU expression is highly induced during *in vivo* infection and responsible for virulence and Hcp1 secretion in avian pathogenic *Escherichia coli*. *Front Microbiol* **5**, 588 (2014).

8. Engstrom, M.D., Alteri, C.J. & Mobley, H.L. A conserved PapB family member, TosR, regulates expression of the uropathogenic *Escherichia coli* RTX nonfimbrial adhesin TosA while conserved LuxR family members TosE and TosF suppress motility. *Infect Immun* **82**, 3644-56 (2014).

9. Johnson, T.J. *et al.* Identification of minimal predictors of avian pathogenic *Escherichia coli* virulence for use as a rapid diagnostic tool. *J Clin Microbiol* **46**, 3987-96 (2008).

10. Trapnell, C., Pachter, L. & Salzberg, S.L. TopHat: discovering splice junctions with RNA-Seq. *Bioinformatics* **25**, 1105-11 (2009).

11. Mortazavi, A., Williams, B.A., McCue, K., Schaeffer, L. & Wold, B. Mapping and quantifying mammalian transcriptomes by RNA-Seq. *Nat Methods* **5**, 621-8 (2008).

12. Wu, Z. *et al.* The *Streptococcus suis* transcriptional landscape reveals adaptation mechanisms in pig blood and cerebrospinal fluid. *RNA* **20**, 882-98 (2014).

13. Trapnell, C. *et al.* Differential analysis of gene regulation at transcript resolution with RNA-seq. *Nat Biotechnol* **31**, 46-53 (2013).

14. Kansal, R. *et al.* Transcriptional modulation of enterotoxigenic *Escherichia coli* virulence genes in response to epithelial cell interactions. *Infect Immun* **81**, 259-70 (2013).

15. Cai, W. *et al.* A novel two-component signaling system facilitates uropathogenic *Escherichia coli*'s ability to exploit abundant host metabolites. *PLoS Pathog* **9**, e1003428 (2013).

16. Livak, K.J. & Schmittgen, T.D. Analysis of relative gene expression data using real-time quantitative PCR and the 2(-Delta Delta C(T)) Method. *Methods* **25**, 402-8 (2001).

17. Uppal, S., Shetty, D.M. & Jawali, N. Cyclic AMP receptor protein regulates *cspD*, a bacterial toxin gene, in *Escherichia coli*. *J Bacteriol* **196**, 1569-77 (2014).

18. Schneider, G. *et al.* The pathogenicity island-associated K15 capsule determinant exhibits a novel genetic structure and correlates with virulence in uropathogenic *Escherichia coli* strain 536. *Infect Immun* **72**, 5993-6001 (2004).

19. King, J.E., Aal Owaif, H.A., Jia, J. & Roberts, I.S. Phenotypic Heterogeneity in Expression of the K1 Polysaccharide Capsule of Uropathogenic *Escherichia coli* and Downregulation of the Capsule Genes during Growth in Urine. *Infect Immun* **83**, 2605-13 (2015).

20. Frosch, M., Gorgen, I., Boulnois, G.J., Timmis, K.N. & Bitter-Suermann, D. NZB mouse system for production of monoclonal antibodies to weak bacterial antigens: isolation of an IgG antibody to the polysaccharide capsules of *Escherichia coli* K1 and group B *meningococci*. *Proc Natl Acad Sci U S A* **82**, 1194-8 (1985).

21. Bak, G., Han, K., Kim, D. & Lee, Y. Roles of *rpoS*-activating small RNAs in pathways leading to acid resistance of *Escherichia coli*. *Microbiologyopen* **3**, 15-28 (2014).

22. Seo, S.W., Kim, D., O'Brien, E.J., Szubin, R. & Palsson, B.O. Decoding genome-wide GadEWX-transcriptional regulatory networks reveals multifaceted cellular responses to acid stress in *Escherichia coli*. *Nat Commun* **6**, 7970 (2015).

23. Zhuge, X. *et al.* Characterization and functional analysis of AatB, a novel autotransporter adhesin and virulence factor of avian pathogenic *Escherichia coli*. *Infect Immun* **81**, 2437-47 (2013).

**Supplementary Figure S1.**


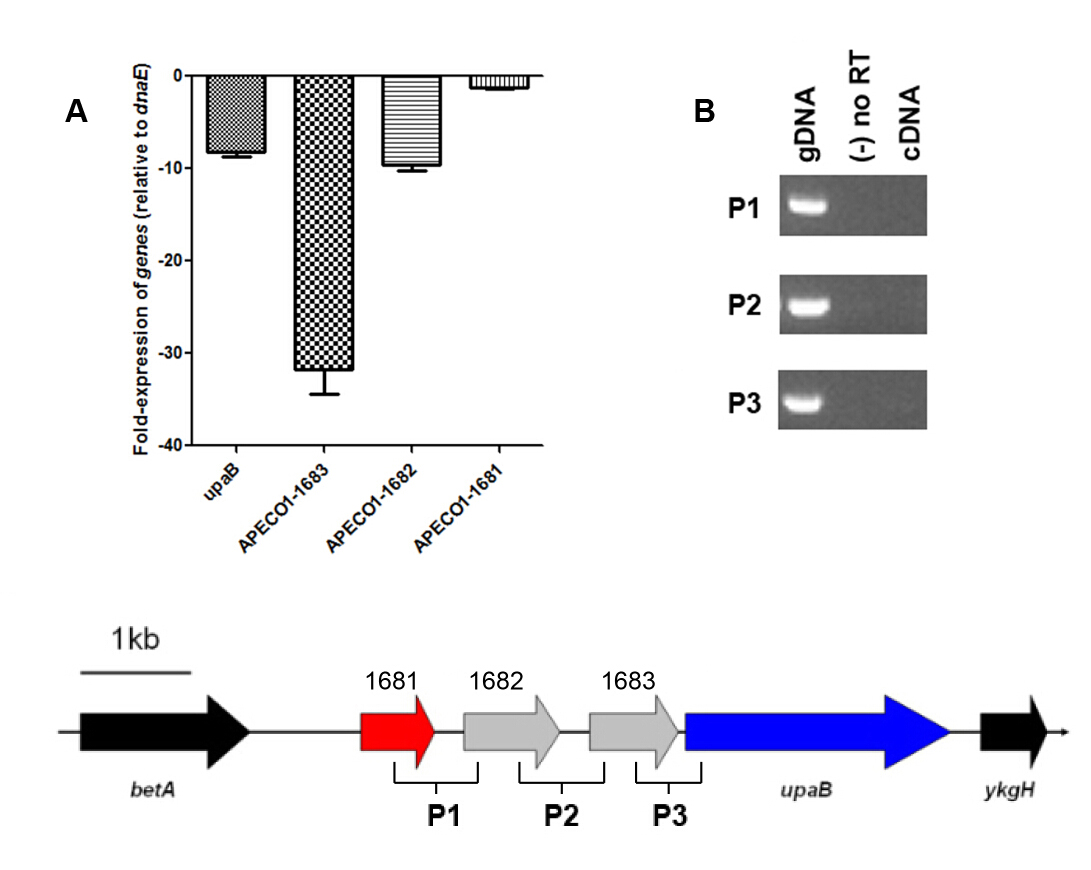


(A) RNA level of four ORFs in DE205B. RT-PCR data represent mean relative expression of three biological replicates. (B) The co-transcription test for intergenic regions of the four ORFs of UpaB cluster. RNA of DE205B was reverse transcribed to cDNA. The RNA that was not reverse transcribed acted as a negative control.

**Supplementary Figure S2.**


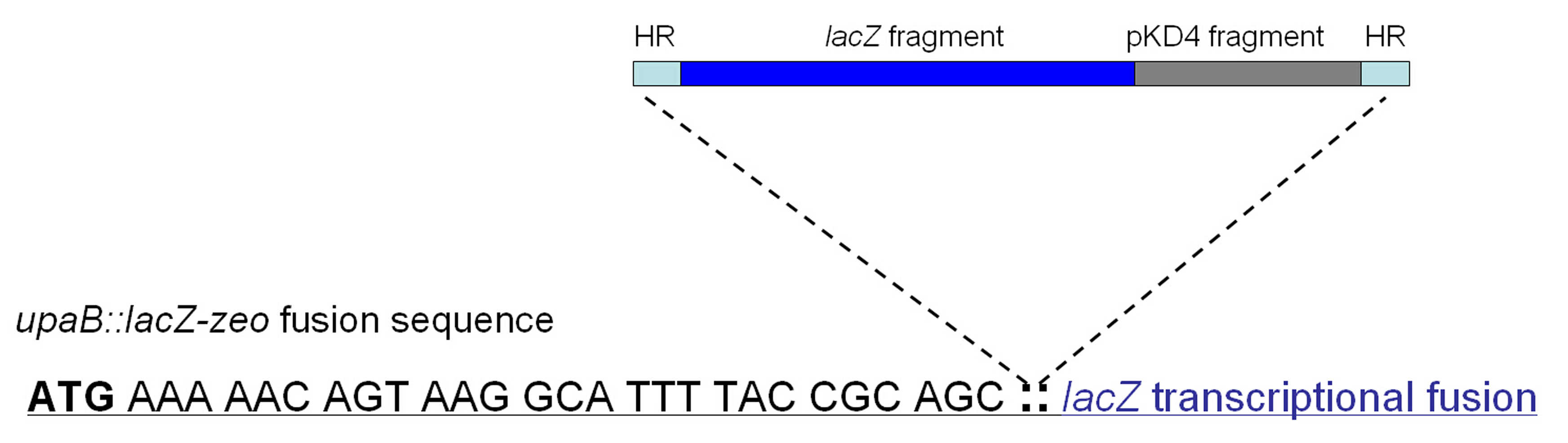


Schematic illustrating construction of the *upaB::lacZ-zeo* transcriptional reporter fusion in DE205BΔ*lacI-Z*. The fusion PCR was performed to connect the two fragments: one was the *lacZ* product containing the regions of homology to 5’ end of *upaB* sequence, and the other for kanamycin resistance cassettes contained the regions of homology to 3’ end of *upaB* sequence.

**Supplementary Figure S3.**


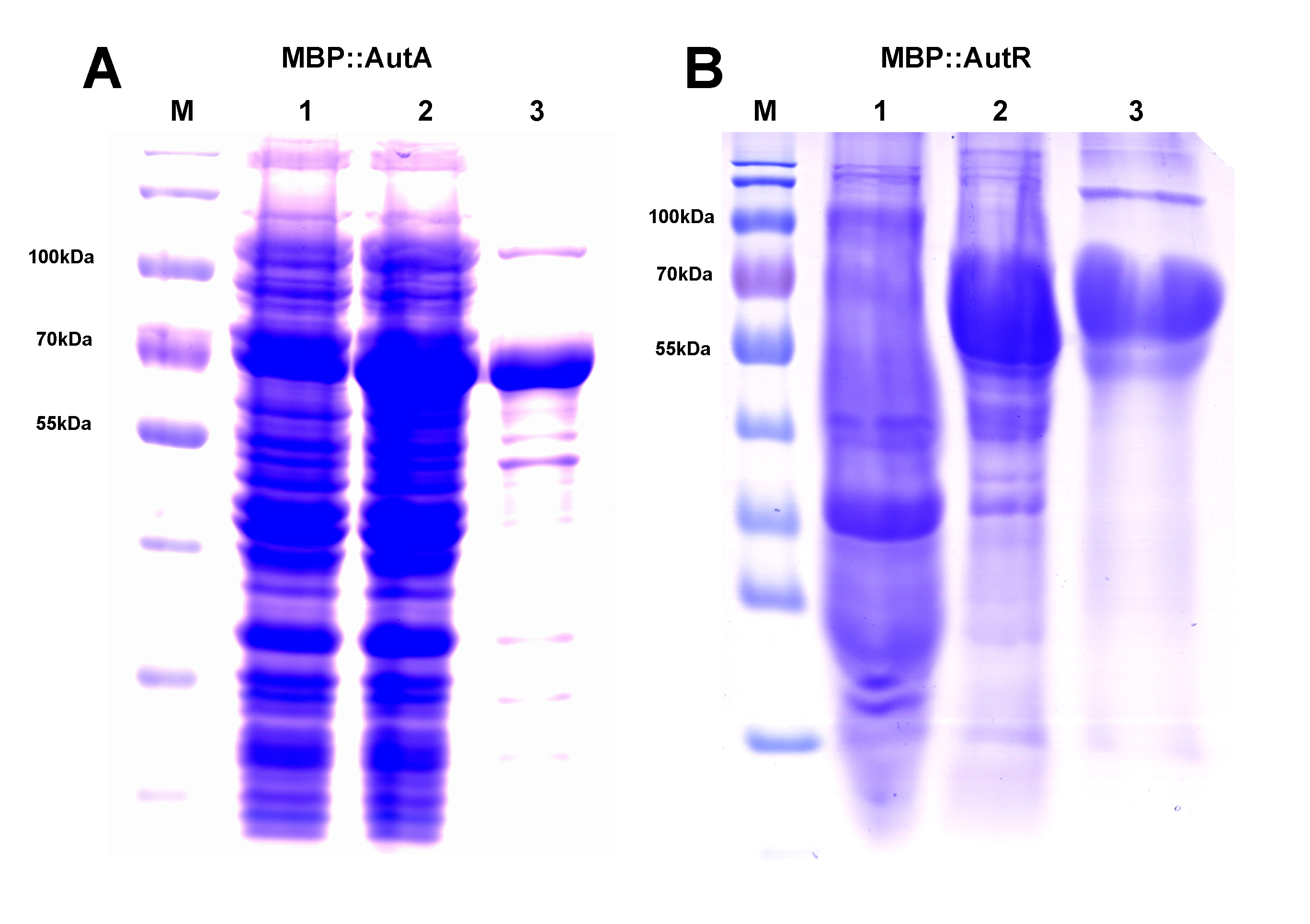


Purification of MBP::AutA and MBP::AutA fusion proteins expressed in *E. coli* BL21(DE3). The whole ORF of *autA* and *autR* was cloned into pCold-malE. Proteins from the total bacteria extracts (lanes 1 and 2) and the purified MBP::AutA and MBP::AutA (lane 1) were separated on an SDS-PAGE with Coomassie, respectively. Lane M, protein marker.

**Supplementary Figure S4.**


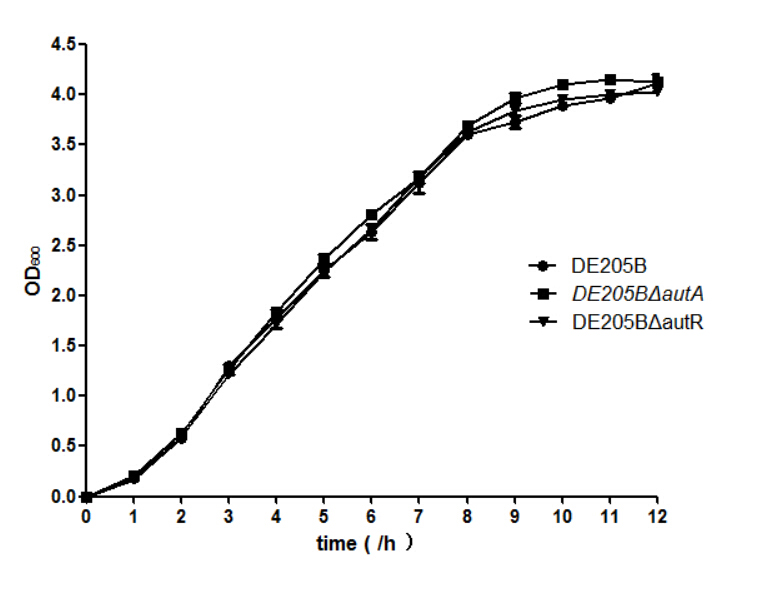


The growth kinetics of DE206B and the mutants (DE205BΔ*autA* and DE205BΔ*autR*) for routine cultured condition.

**Supplementary Figure S5.**


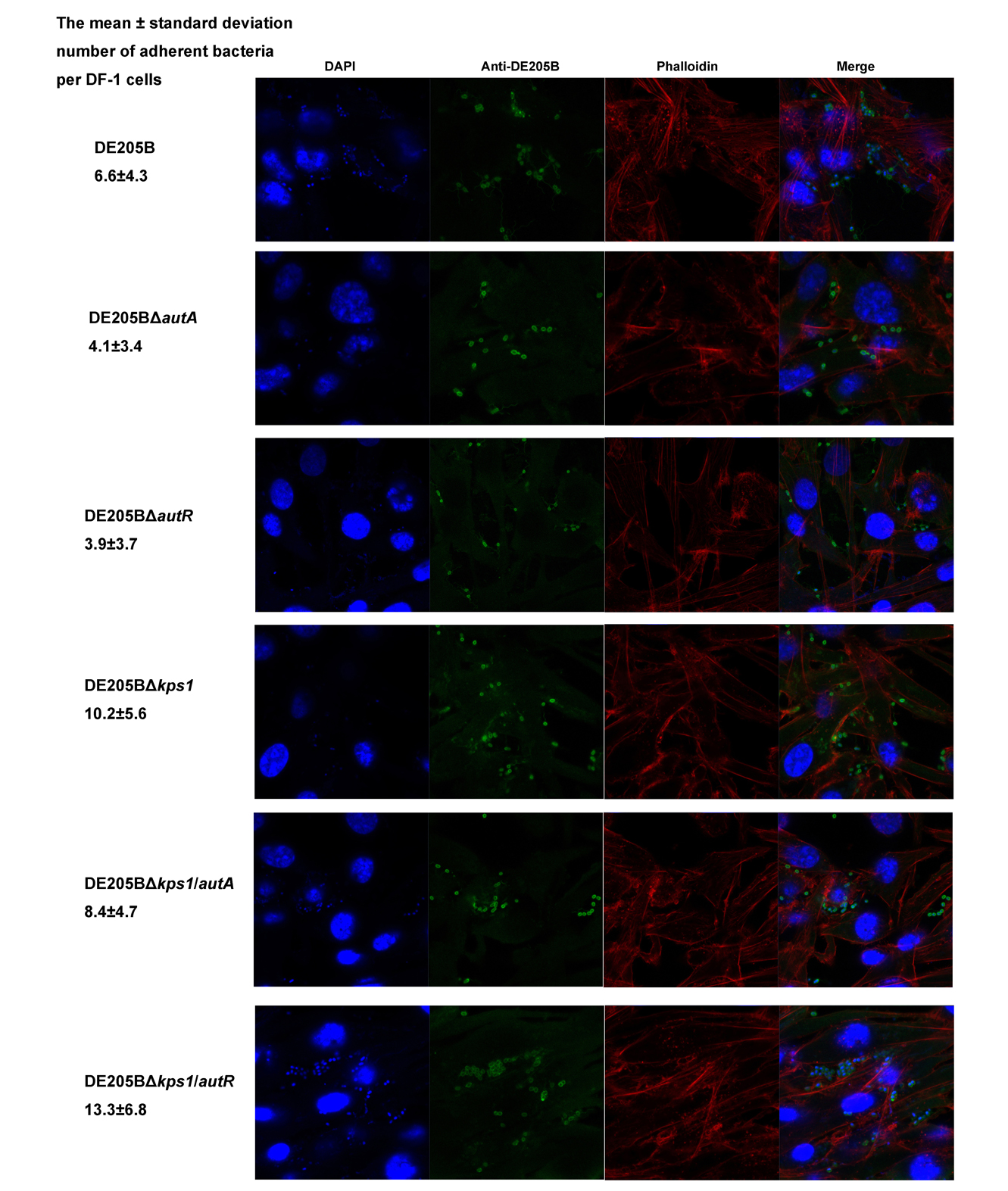


The imaging of the bacteria counting in infected DF-1 cells. The immunofluorescent imaging of bacteria was detected using a Zeiss LSM-510 META confocal laser scanning microscope. The number of bacteria adhering to DF-1 cells could be directly counted from immunofluorescent imaging. The mean ± standard deviation number of adherent bacteria per DF-1 cells was shown for each strain.

**Supplementary Figure S6.**


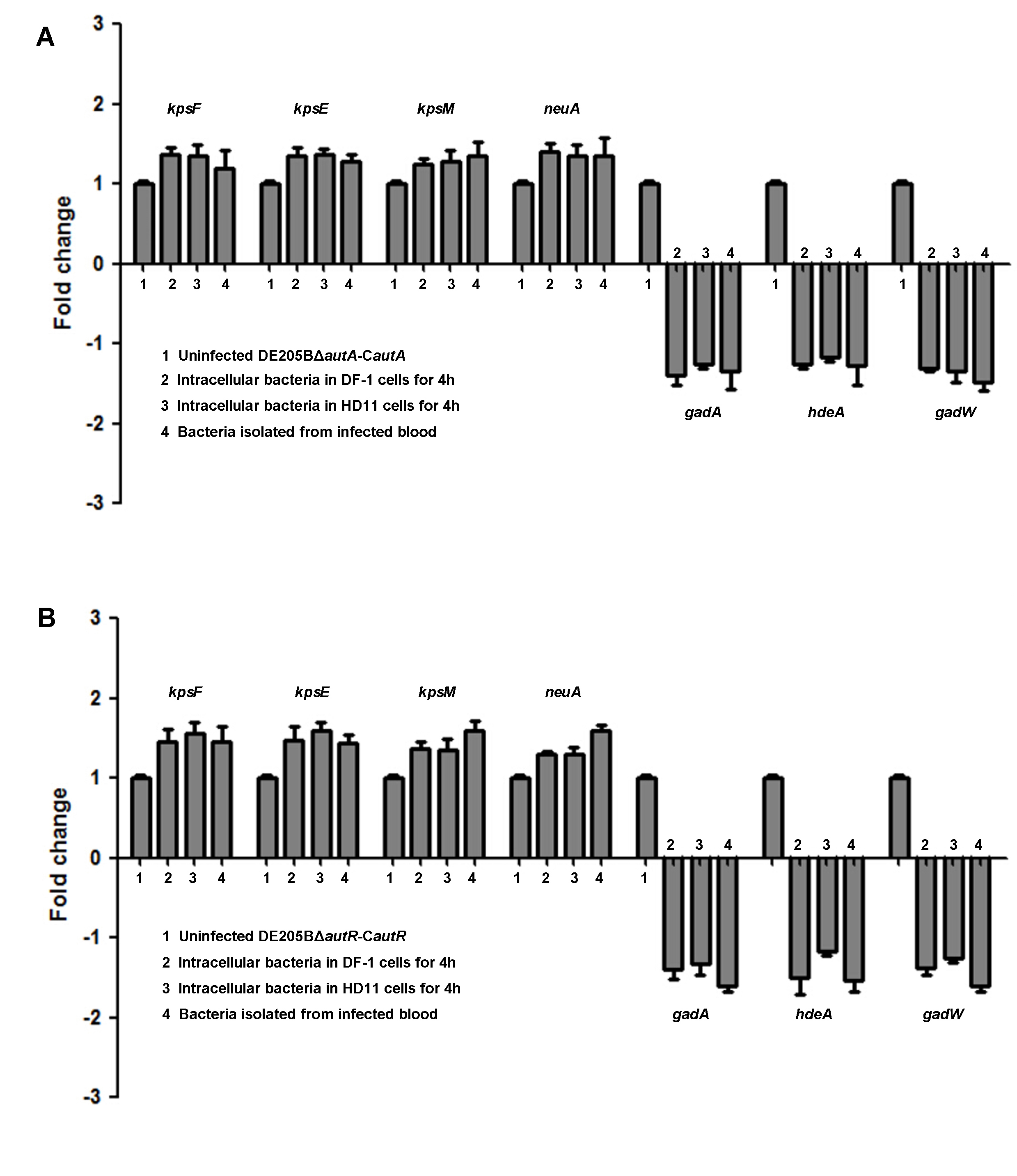


Expression profile of K1 capsule and acid resistance systems among wild-type DE205B, DE205BΔ*autA*-C*autA* and DE205BΔ*autR*-C*autR* during infection *in vitro* and serum resistance for sepsis *in vivo*. qRT-PCR was performed measure the expression of these genes. Statistical significance analysis was performed using two-way ANOVA (*, *P*<0.05).

**Supplementary Table S1.** Transcriptome analysis showed that regulators AutA and AutR coherently affected hundreds of genes expression

**DE205B VS DE205B****Δ*autA***

**DE205B VS DE205BΔ*autR***

**Supplementary Table S2.** RT-PCR result of the 50 genes for expression change among wild-type DE205B, DE205BΔ*autA* and DE205BΔ*autR*. qRT-PCR was performed measure the expression of these genes. Statistical significance analysis was performed using two-way ANOVA (*, *P*<0.05).

| gene_id | genes | log_2_(fold change) for DE205BΔ*autA* relative to DE205B (-,down;+,up) | log_2_(fold change) for DE205BΔ*autR* relative to DE205B (-,down;+,up) |
| --- | --- | --- | --- |
| L282_0142 | *wzy* | (+) 2.834 | (+) 2.763 |
| L282_0145 | *wzx* | (+) 2.412 | (+) 2.517 |
| L282_0237 | *mlrA* | (+) 3.981 | (+)4.126 |
| L282_0406 | *lrhA* | (+) 2.674 | (+) 2.441 |
| L282_0524 | *dsdC* | (+) 2.710 | (+) 2.637 |
| L282_0856 | *proX* | (+) 3.632 | (+)3.757 |
| L282_0991 | *fucR* | (+) 3.155 | (+)3.012 |
| L282_1065 | *ygeV* | (+) 3.687 | (+)3.34 |
| L282_1101 | *gcvH* | (+) 4.028 | (+)3.879 |
| L282_1172 | *kpsF* | (+) 4.349 | (+) 4.403 |
| L282_1173 | *kpsE* | (+) 3.836 | (+) 3.912 |
| L282_1178 | *neuS* | (+) 4.270 | (+) 4.087 |
| L282_1180 | *neuE* | (+) 4.031 | (+) 4.114 |
| L282_1185 | *kpsT* | (+)5.370 | (+)5.240 |
| L282_1231 | *hybA* | (+) 2.614 | (+)2.566 |
| L282_1260 | *ygiV* | (-) 2.568 | (-)2.57 |
| L282_1359 | *tdcC* | (+) 2.440 | (+)2.638 |
| L282_1752 | *yhiF* | (-) 5.138 | (-) 4.787 |
| L282_1762 | *chuV* | (-) 4.342 | (-)4.313 |
| L282_1763 | *yhiD* | (-) 7.199 | (-)7.467 |
| L282_1765 | *hdeA* | (-) 6.787 | (-)7.65 |
| L282_1767 | *gadE* | (-) 7.216 | (-)7.353 |
| L282_1770 | *mdtF* | (-) 5.720 | (-) 5.214 |
| L282_1772 | *gadX* | (-) 3.820 | (-) 4.375 |
| L282_1773 | *gadA* | (-) 6.791 | (-) 7.334 |
| L282_1821 | *cspA* | (-) 6.121 | (-)5.892 |
| L282_1999 | *atpC* | (+) 2.750 | (+)2.801 |
| L282_2002 | *atpA* | (+) 2.813 | (+)2.663 |
| L282_2004 | *atpF* | (+) 2.882 | (+)2.774 |
| L282_2025 | *hdfR* | (+) 2.503 | (+)2.49 |
| L282_2203 | *glpK* | (-) 3.734 | (-)3.76 |
| L282_2227 | *gldA* | (+) 3.168 | (+)3.246 |
| L282_2527 | *aatA* | (-) 3.255 | (-)3.288 |
| L282_2575 | *ecnB* | (-) 4.462 | (-)4.471 |
| L282_2878 | *caiF* | (+) 3.337 | (+)3.388 |
| L282_2909 | *araC* | (+) 2.960 | (+)3.112 |
| L282_3434 | *cstA* | (+) 5.388 | (+)5.129 |
| L282_3550 | *sucC* | (+) 3.677 | (+)3.747 |
| L282_3555 | *cydA* | (+) 3.417 | (+)3.576 |
| L282_3629 | *glnH* | (+) 4.766 | (+)4.891 |
| L282_3744 | *cspD* | (+) 3.858 | (+)3.583 |
| L282_3842 | *hyaC* | (-) 4.225 | (-)4.302 |
| L282_3846 | *appC* | (-) 4.512 | (-)4.61 |
| L282_4164 | *ftnA* | (+) 3.362 | (+)3.383 |
| L282_4173 | *otsB* | (-) 4.402 | (-)4.487 |
| L282_4556 | *gadB* | (-) 7.743 | (-) 7.683 |
| L282_4557 | *gadC* | (-) 7.288 | (-) 7.529 |
| L282_4651 | *uspF* | (+) 3.356 | (+)3.372 |
| L282_4744 | *ompW* | (+) 2.481 | (+)2.127 |
| L282_4758 | *oppA* | (+) 5.346 | (+)5.104 |

**Supplementary Table S3.** Bacterial strains and plasmids used in this study.

| **Bacterial strains and plasmids** | **Genotype or relevant characteristics** | **Source or Reference** |
| --- | --- | --- |
| **Bacterial strains** |  |  |
| *E. coli* DH5α | Plasmid propagation strain | Invitrogen |
| *E. coli* BL21 (DE3) | F-, *ompT, hsdS (r_B_^-^ m_B_^-^) gal, dcm* (DE3) | TIANGEN |
| DE205B | O2:K1; ST complex 95, ST140; phylogroup B2 | [^1-3^](#_ENREF_1) |
| DE205BΔ*upaB* | Adhesin gene *upaB* deletion in DE205B | 1 |
| DE205BΔ*autA* | *autA* deletion in DE205B | This study |
| DE205BΔ*autR* | *autR* deletion in DE205B | This study |
| DE205BΔ*autA/autR* | Parallel deletion of *autA* and *autR* in DE205B | This study |
| DE205BΔ*autA-*C*autA* | DE205BΔ*autA* with plasmid pSTV28-*autA* | This study |
| DE205BΔ*autR-*C*autR* | DE205BΔ*autR* with plasmid pSTV28-*autR* | This study |
| DE205BΔ*autA/autR*-C*autA* | DE205BΔ*autA/autR* with plasmid pSTV28-*autA* | This study |
| DE205BΔ*autA/autR*-C*autR* | DE205BΔ*autA/autR* with plasmid pSTV28-*autR* | This study |
| DE205BΔ*lacI-Z* | Parallel deletion of *lacI* and *lacZ* in DE205B | This study |
| DE205BΔ*lacI-Z upaB::lacZ-zeo* | The *upaB::lacZ-zeo* transcriptional reporter fusion in DE205BΔ*lacI-Z* | This study |
| DE205BΔ*lacI-Z/autA upaB::lacZ-zeo* | *autA* deletion in DE205BΔ*lacI-Z upaB::lacZ-zeo* | This study |
| DE205BΔ*lacI-Z/autR* *upaB::lacZ-zeo* | *autR* deletion in DE205BΔ*lacI-Z upaB::lacZ-zeo* | This study |
| DE205BΔ*lacI-Z/autA/autR upaB::lacZ-zeo* | Parallel deletion of *autA* and *autR* in DE205BΔ*lacI-Z upaB::lacZ-zeo* | This study |
| DE205BΔ*lacI-Z/autA-CautA upaB::lacZ-zeo* | DE205BΔ*lacI-Z/autA upaB::lacZ-zeo* with plasmid pSTV28-*autA* | This study |
| DE205BΔ*lacI-Z/autR-CautR upaB::lacZ-zeo* | DE205BΔ*lacI-Z/autR* *upaB::lacZ-zeo* with plasmid pSTV28-*autR* | This study |
| DE205BΔ*lacI-Z/autA/autR-CautA upaB::lacZ-zeo* | DE205BΔ*lacI-Z/autA/autR upaB::lacZ-zeo* with plasmid pSTV28-*autA* | This study |
| DE205BΔ*lacI-Z/autA/autR-CautR upaB::lacZ-zeo* | DE205BΔ*lacI-Z/autA/autR upaB::lacZ-zeo* with plasmid pSTV28-*autR* | This study |
| DE205BΔ*kps1* | *kpsFEDUC* deletion in DE205B | This study |
| DE205BΔ*kps1*-C*kps* | DE205BΔ*kps1* with plasmid pGEN-*kpsFEDUC* | This study |
| DE205BΔ*kps1*/*autA* | *autA* deletion in DE205BΔ*kps1* | This study |
| DE205BΔ*kps1*/*autR* | *autR* deletion in DE205BΔ*kps1* | This study |
| DE205BΔ*kps1*/*autA*-C*autA* | DE205BΔ*kps1*/*autA* with plasmid pSTV28-*autA* | This study |
| DE205BΔ*kps1*/*autR*-C*autR* | DE205BΔ*kps1*/*autA* with plasmid pSTV28-*autR* | This study |
|  |  |  |
| ***Plasmids*** |  |  |
| pCold-*malE* | fusing *malE* coding sequence into the pColdⅠ(TaKaRa) | This study |
| pCold-*malE*/*autA* | pCold-*malE* carrying *autA* gene | This study |
| pCold-*malE*/*autR* | pCold-*malE* carrying *autR* gene | This study |
| pMAL-c2X | expression vector under the control of P_tac_ | New England Biolabs |
| pGEN-MCS | A low copy plasmid for complementation | [^4^](#_ENREF_4) |
| pGEN-*kpsFEDUC* | pGEN-MCS derivative harboring K1 capsule operon *kpsFEDUC* and its putative promoter | This study |
| pSTV28 | A medium-copy plasmid | Takara |
| pSTV28-*autA* | pSTV28 carrying *autA* coding region and its putative promoter | This study |
| pSTV28-*autR* | pSTV28 carrying *autR* coding region and its putative promoter | This study |
| pKD4 | template for λ-Red Kan^r^ cassette | [^5^](#_ENREF_5) |
| pCP20 | encodes FLP recombinase for removal of resistance cassette | [^5^](#_ENREF_5) |
| pKD46 | λ-Red recombinase expression | [^5^](#_ENREF_5) |

**References**

1. Zhu-Ge, X.K. *et al.* The effects of *upaB* deletion and the double/triple deletion of *upaB*, *aatA*, and *aatB* genes on pathogenicity of avian pathogenic *Escherichia coli*. *Appl Microbiol Biotechnol* **99**, 10639–10654 (2015).

2. Zhu Ge, X. *et al.* Comparative genomic analysis shows that avian pathogenic *Escherichia coli* isolate IMT5155 (O2:K1:H5; ST complex 95, ST140) shares close relationship with ST95 APEC O1:K1 and human ExPEC O18:K1 strains. *PLoS One* **9**, e112048 (2014).

3. Zhuge, X. *et al.* Characterization and functional analysis of AatB, a novel autotransporter adhesin and virulence factor of avian pathogenic *Escherichia coli*. *Infect Immun* **81**, 2437-47 (2013).

4. Lane, M.C., Alteri, C.J., Smith, S.N. & Mobley, H.L. Expression of flagella is coincident with uropathogenic *Escherichia coli* ascension to the upper urinary tract. *Proc Natl Acad Sci U S A* **104**, 16669-74 (2007).

5. Datsenko, K.A. & Wanner, B.L. One-step inactivation of chromosomal genes in *Escherichia coli* K-12 using PCR products. *Proc Natl Acad Sci U S A* **97**, 6640-5 (2000).

**Supplementary Table S4.** Oligonucleotide sequences used as PCR primers in this study.

| **Primers** | **Sequence (5'-3')** |
| --- | --- |
| **General PCR for cloning** |  |
| pGEN-*kpsFEDUC* 1-F | CCGGAATTCCACCTCCATGAGACATTGCGACT |
| pGEN-*kpsFEDUC* 1-R | CATGCATGCTGGGGTGTCGACG |
| pGEN-*kpsFEDUC* 2-F | CATGCATGCCTGGAAAAAGTGCG |
| pGEN-*kpsFEDUC* 2-R | ATTTGCGGCCGCGCGCCTCTTATGATAAAGTTGTGTG |
| pSTV28-*autA*-F | CCCAAGCTTTTCTAGCTAAAGTAAAATAACCCCA |
| pSTV28-*autA*-R | CGCGGATCCGTAGTAACAACGCTGCTGTGTCATA |
| pSTV28-*autR*-F | CCGGAATTCGACCAGGATTGTTATGACACAGCAG |
| pSTV28-*autR*-R | CCCAAGCTTTTCTGGTAACTAATTTCAATTTCCC |
| pCold-*malE*/*autA*-F | CGAGCTCATGAAGAAGCCTCTGGTTATTATC |
| pCold-*malE*/*autA*-R | CCGCTCGAGTAACAATCCTGGTCTTTTTTTAGATAT |
| pCold-*malE*/*autR*-F | CCGCTCGAGATGCGTTTCGTTCTTTTCTGTC |
| pCold-*malE*/*autR*-R | CCGGAATTCTTTATATTTATGCAGAGATAAAGCCT |
| ***For lacZ fusion*^a^** |  |
| *lacZ*-F | AAATTAGTTACCAGAATGGAGAATTTCTTCATGAAAAACAGTAAGGCATTTTACCGCAGCactatgattacggattctctggc |
| *lacZ*-R | TTATTTTTGACACCAGACCA |
| pKD4-F | TGGTCTGGTGTCAAAAATAAGTGTAGGCTGGAGCTGCTTC |
| pKD4-R | GCAGCATACTGCTGCCTGCAGGGGGTATAACAGTCGACAGGGGAACCGACTGCTTTATTCcatatgaatatcctccttag |
| Fusion-F | AAATTAGTTACCAGAATGGAGAATTTCTTC |
| Fusion-R | GCAGCATACTGCTGCCTGCAG |
| **For Deletion^a^** |  |
| Del-*autA*-F | ATTTTGAATCAAAATGTCAGGTTTTGTAGCAATGGCGTGGACGAGTCTGAgtgtaggctggagctgcttc |
| Del-*autA*-R | GTAGCGAGTCCGTATGCGCCGCAGTGGGTGGTAGTAACAACGCTGCTGTGcatatgaatatcctccttag |
| Del-*autR*-F | GCTGTACAGCCTGTACAGCGTGCTGGCGTACTGACATCTGTGAGGGAAGGgtgtaggctggagctgcttc |
| Del-*autR*-R | TGCATTTCCATCACCGTGAAAATTATTGCTGCTCAGCAGCAGGGCTTCCCcatatgaatatcctccttag |
| Del-*lacI-Z*-F | AAAACCTTTCGCGGTATGGCATGATAGCGCCCGGAAGAGAGTCAATTCAGGGTGGTGAATgtgtaggctggagctgcttc |
| Del-*lacI-Z*-R | ATAGTACATAATGGATTTCCTTACGCGAAATACGGGCAGACATGGCCTGCCCGGTTATTAcatatgaatatcctccttag |
| Del-*kpsFEDUC*-F | AATTATAGTGGGTTCGGGTTTGTTGTGACTGTGGCATTATTTCCGTGCAAAGGAGCTGATgtgtaggctggagctgcttc |
| Del-*kpsFEDUC*-R | TTGAAGAGGATGGAAATGATTTTTTGGCTACTTAAAATTCAAAAGATATTGACTTGAAATcatatgaatatcctccttag |
| **For Multiplex PCR genotyping** |  |
| MultiP PCR-Integrase-F | AAGTCTACAGGCTGATGGATGC |
| MultiP PCR-Integrase-R | GCATTACTGGCTGTGTATCTGA |
| MultiP PCR-*autA*-F | TTCTAGCTAAAGTAAAATAACCCCA |
| MultiP PCR-*autA*-R | GTAGTAACAACGCTGCTGTGTCATA |
| MultiP PCR-*autR*-F | ATGCGTTTCGTTCTTTTCTGTC |
| MultiP PCR-*autR*-R | TTCTGGTAACTAATTTCAATTTCCC |
| MultiP PCR-*upaB*-F | ACTGATAGCACGGTATCAACTGA |
| MultiP PCR-*upaB*-R | TTTATGCGTGTTTTTTACGAA |
| **For EMSA** |  |
| P*_upaB_*-F | TTTTCACGGTGATGGAAATGC |
| P*_upaB_*-R | GCTGCGGTAAAATGCCTTACTG |
| *upaB*-F | TAATGTTACTTTTGGCGGTCACGAT |
| *upaB*-R | CGCCATTAACAACAACATCGCCAGT |
| ***In vitro* transcription** |  |
| P*_upaB_*-*upaB*-F | GGATAAGACAGATATGGCTTGCCGA |
| P*_upaB_*-*upaB*-R | CATTGAGCGTTAAATTATTTCCCGT |
| P*_tac_*-*malE*-F | GAAGCTGTGGTATGGCTGTGCA |
| P*_tac_*-*malE*-R | ATACTTGAACGCATAACCCCCG |
| *upaB*-RT-F | CCGCATCAACGGTAAACCA |
| *upaB*-RT-R | ATCGCCAGTTGAATCAATGACC |
| *malE*-RT-F | CGGTCTCGCTGAAGTCGGTAA |
| *malE*-RT-R | TGCCGCAACCTGTGGGAAT |
| **For co-transcription** |  |
| Opr-Integrase/*autA*-F | CCTGTGGTTTTGCTCTGGCTGAC |
| Opr-Integrase/*autA*-R | ATGAGAGACTCCAGTGCCAGCCT |
| Opr-*autA*/*autR*-F | TCGACTCACTCAGTATGTGCAGAA |
| Opr-*autA*/*autR*-F | GCGTAACTCCTCTTCTGTTCTGAT |
| Opr-*autR*/*upaB*-F | GTGAAAACGGAAATACCAACAGG |
| Opr-*autR*/*upaB*-R | ACGGCTGTAATCTTGTTATCACC |
| **For RT-PCR** |  |
| Integrase-qPCR-F | GGAAACGCCGCCCTATCA |
| Integrase-qPCR-R | AGTAGTCGGGTATCGACACCAT |
| *autA*-qPCR-F | CTGGGAACACTGAGCGATTT |
| *autA*-qPCR-R | AGTCGATGAGCGGAATGTTG |
| *autR*-qPCR-F | TACAGTGCGCAGTTTCTCAA |
| *autR*-qPCR-R | TGACATGCTGTCCGGTATTC |
| *upaB*-qPCR-F | CGCTCTTACTGGGAAGGTAATG |
| *upaB*-qPCR-R | TGCCTTTCCAGACTGAACTATC |

- Underlined are restriction cutting sites;
- Capital letters represent homologous fragments of the deleted genes.
